# Mathematical modelling and analysis of the adaptive dynamics in mosquito populations: uniform persistence of malaria infection

**DOI:** 10.1007/s00285-025-02206-z

**Published:** 2025-03-26

**Authors:** Bassirou Diop, Arnaud Ducrot, Ousmane Seydi

**Affiliations:** 1 École Polytechnique de Thiès, BP A10, Thiès, Senegal; 2 Université Le Havre Normandie, Normandie Univ., LMAH UR3821, 76600 Le Havre, France

**Keywords:** Malaria transmission, Integro-differential equations, Uniform persistence, Perturbation, Evolutionary epidemiology, 92D30, 35Q92, 45K05, 35R09

## Abstract

In this work, we present a mathematical model for the spread of malaria, incorporating key factors such as human populations, mosquito behavior, and the mosquitoes’ plasticity and adaptation to control measures like widespread insecticide-treated mosquito nets and indoor residual spraying. Through analysis of the model, we identify and describe the convergence and persistence properties of the solutions, leveraging a small parameter that represents the interactions between mosquitoes in relation to their activity patterns. In our analysis, we extend some ideas from the theory of uniform persistence to the case of semiflow without dissipativity.

## Introduction

Malaria is an infectious disease caused by a parasite belonging to the *Plasmodium* genus. It is transmitted by the bites of infected mosquitoes of the *Anopheles* genus. This disease represents a major public health issue in many tropical and subtropical regions, notably in sub-Saharan Africa, Southeast Asia, and South America (Organization 2023), causing over 608,000 deaths in 2022, with more than $76\%$ of these being children under 5 years old. Therefore, enhancing our understanding of malaria dynamics through mathematical modeling is crucial for developing effective strategies to combat the disease and guide decision-makers.

The mathematical modeling of vector-borne diseases began with the work of Ross (1911) in 1911, where he presented the first mathematical model describing the transmission of malaria. Subsequently, Macdonald (1957) extended Ross’s model and introduced the concept of the basic reproduction number. Based on these advances, the Ross-Macdonald model has experienced rapid development over the years by researchers. We refer for example to Aron and May (1982), Cai et al. (2017), Dietz et al. (1974), Gao and Ruan (2012), Gupta et al. (1994), Magal et al. (2018), Ngwa and Shu (2000) and the references therein. Specifically, the evolutionary dynamics of mosquitoes have been studied in Chitnis et al. (2012), Ngwa and Shu (2000), Lutambi et al. (2013) while the host-vector transmission of malaria has been considered in Feng and Castillo-Chavez (2006), Richard et al. (2022), Mandal et al. (2013), Demasse and Ducrot (2013), Mandal et al. (2011), Ngwa and Shu (2000), Beck et al. (1984). Several mathematical models considering spatial dynamics (Xu and Zhang 2015), time since infection (Richard et al. 2022), immunity (Richard et al. 2022; Aron 1988; Qu et al. 2023), and temperature (Beck-Johnson et al. 2013; Ewing et al. 2016; Lou and Zhao 2010), are available in the literature and may be used by decision makers to control the disease.

Over the past two decades, the use of insecticide-treated mosquito nets and indoor residual spraying, combined with other malaria control methods, has helped to reduce infection in various parts of the world. However, recent researches (Gatton et al. 2013; Sangbakembi-Ngounou et al. 2022; Yohannes and Boelee 2012; Moiroux et al. 2012; Hickner et al. 2022; Govella et al. 2023; Fornadel et al. 2010) on mosquito dynamics has shown that the use of these methods leads to physiological and behavioral changes in mosquitoes. These changes can be refereed as the plasticity of mosquitoes. In Ferreira et al. (2017), the authors argue that the changes in the biting and resting behaviors of the primary malaria vectors observed in recent years are attributable to the selective pressure resulting from the widespread and prolonged use of insecticide-treated mosquito nets and indoor residual spraying. This behavioral resistance significantly impacts the success of malaria control and thus promotes the development of the parasite. Consequently, research on mosquito dynamics through mathematical modeling becomes a crucial issue. There is an important literature on this topic, using deterministic and/or stochastic mathematical models. We refer the reader for instance to Ferreira et al. (2017), Li et al. (2019), Djidjou-Demasse et al. (2020), Richard et al. (2022), Singh et al. (2023) and the references cited therein.

In Djidjou-Demasse et al. (2020), Li et al. (2019), Richard et al. (2022), various approaches using deterministic models have been proposed to understand and control malaria transmission. These models take into account seasonality in the dynamics of mosquito populations, malaria transmission, and waning immunity. They also highlight the behavioral plasticity of mosquitoes according to their age. We refer to Ferreira et al. (2017); Singh et al. (2023), where stochastic models have been used to study the impact of insecticide-treated bed nets on mosquito fitness, as well as the uncertainties and stochastic variations in malaria transmission.

In this work we consider the mathematical model below to describe the interactions between host and vector populations, that is between mosquitoes and humans, by incorporating behavioral changes in the vector populations, due to adaptations. Moreover, this work focuses on the timing of host-vector contact, specifically the hour of mosquito bites, which we denote by *x*. Throughout this study, *x* is referred to as a phenotypic trait. This choice is supported by quantitative trait locus (QTL) analysis of *Culex pipiens* strains with contrasting feeding behaviors, as demonstrated in Hickner et al. (2022). Their study identifies a genetic basis for variation in feeding times, reinforcing the consideration of biting time as a significant phenotypic trait in mosquito behavior. The model we consider is given by the following formulation

1.1 $$\begin{aligned} \text {(Humans)}{\left\{ \begin{array}{ll} \dfrac{\textrm{d}S_h(t)}{\textrm{d}t}=\Lambda -\dfrac{S_h(t)}{N_h(t)}\int _0^{1} p(y)I_m(t,y)\textrm{d}y+\gamma _1I_h(t)-\mu _hS_h(t),\\ \dfrac{\textrm{d}I_h(t)}{\textrm{d}t}=\dfrac{S_h(t)}{N_h(t)}\int _0^{1} p(y)I_m(t,y)\textrm{d}y -(\gamma _1+\gamma _2)I_h(t)-\mu _hI_h(t), \end{array}\right. }\nonumber \\ \end{aligned}$$

and

1.2 $$\begin{aligned} \text {(Mosquitoes)}{\left\{ \begin{array}{ll} \dfrac{\partial S_m(t,x)}{\partial t}=D\partial _{xx} S_m(t,x)-\sigma (x)\dfrac{I_h(t)}{N_h(t)}S_m(t,x)+bN_m(t,x)\\ \qquad \qquad \qquad \quad -S_m(t,x)\left[ \mu _m(x)+\int _0^{1} K_\epsilon (x-y)N_m(t,y)\textrm{d}y\right] ,\\ \dfrac{\partial I_m(t,x)}{\partial t}=D\partial _{xx} I_m(t,x)+\sigma (x)\dfrac{I_h(t)}{N_h(t)}S_m(t,x)\\ \qquad \qquad \qquad \quad -I_m(t,x)\left[ \mu _m(x)+\int _0^{1}K_\epsilon (x-y)N_m(t,y)\textrm{d}y\right] . \end{array}\right. }\nonumber \\ \end{aligned}$$

The above system is posed for time $t>0$ and supplemented with appropriate nonnegative initial data. As mentioned previously, *x* denotes the hour of mosquito bites, representing a phenotypic trait of the vector population. This trait is subject to adaptive evolution, which is modeled by a Laplace operator. In our modeling approach, we neglect the mutation process in light of recent findings in Govella et al. (2023), which indicate that the heritability of biting time adaptation is low and that changes in biting behavior occur predominantly through selection.

Here for notational simplicity and up to normalization, we assume that $x\in [0,1]$ (instead of a biting hour between 0 and 24 h) and (1.2) is supplemented with periodic boundary conditions.

Next system (1.1)–(1.2) describes the evolutionary dynamics of malaria infection in an environment with high usage of insecticide-treated mosquito nets and indoor residual spraying. Here, recall that $t>0$ represents time, and $x \in [0,1]$ corresponds to the hour of mosquito bites. The quantities $S_h(t)$, $I_h(t)$ denote respectively the densities of susceptible and infected humans at time *t*, while $S_m(t,x)$, $I_m(t,x)$ represent the densities of susceptible and infected mosquitoes, with phenotype trait *x* at time *t*. Additionally, the functions $N_h(t)$ and $N_m(t,x)$ represent the total densities of humans and trait specific mosquitoes at time in the environment, respectively, namely

$$\begin{aligned} N_h(t)=S_h(t)+I_h(t),\;\;N_m(t,x)=S_m(t,x)+I_m(t,x). \end{aligned}$$

As far as the host population is concerned, we assume that susceptible humans are replenished at a constant rate $\Lambda$ and exhibits a natural death rate $\mu _h$. The parameters $\gamma _1$ and $\gamma _2$ correspond to the recovery and additional death rate due to the disease, respectively.

The force of infection from vectors to the hosts takes the form $p(x)S_h(t)/N_h(t)$, where *p*(*x*) denotes the trait specific combined probability of contact between a human and a mosquito and the probability of the disease transmission, while the ratio $S_h(t)/N_h(t)$ corresponds to the fraction of susceptible host individuals. On the other hand, the force of infection from humans to mosquitoes is given by $\sigma (x)I_h(t)/N_h(t)$, where $\sigma (x)$ represents the trait specific probability of contact between a mosquito and a human, combined with the probability of parasite transmission from mosquitoes to humans. The birth rate per individual mosquito is denoted by *b*, and the (trait specific) death rate of both susceptible and infected mosquitoes is given by $\mu _m(x)$.

As mentioned above, the terms $D\partial _{xx} S_m(t,x)$ and $D\partial _{xx} I_m(t,x)$ represent the adaptation in the phenotype trait space of the biting hour in the susceptible and infected mosquito populations. Finally the term $\int _0^{1}K_\epsilon (x-y)N_m(t,y)\textrm{d}y$ models intraspecific competition within the mosquito population. Here we consider that this interaction depends on the hour of mosquito bites, when different mosquitoes try to feed at the same time on limited resources. In other words, mosquitoes with a phenotypic trait *x* compete with those with the trait *y* and we introduce some parameter $\epsilon >0$ to mention that this intraspecific interaction is strong when $y\in [x-\epsilon ,x+\epsilon ]$ (modulo 1) and rather low when the mosquitoes do not feed on the same temporal frame, that is $y\notin [x-\epsilon ,x+\epsilon ]$ (modulo 1). A characteristic function of the interval $[x-\epsilon ,x+\epsilon ]$ has been used in Li et al. (2019). Here to express and consider more general competitions, we assume that the kernel function *K* is defined, for $\epsilon >0$ and $x \in [0,1]$, by:

1.3 $$\begin{aligned} K_\epsilon (x)={\left\{ \begin{array}{ll} \sum \limits _{k\in \mathbb {Z}}\frac{1}{\epsilon }J\left( \frac{x+k}{\epsilon }\right) & \,\text {if}\; \epsilon >0,\\ \sum \limits _{k\in \mathbb {Z}}\delta _k(x)& \,\text {if}\; \epsilon =0, \end{array}\right. } \end{aligned}$$

where $J\in L^1\left( \mathbb {R}; \mathbb {R}^+\right)$ is a given nonnegative and integrable function and $\delta _k$ denotes the Dirac mass at $x=k$.

Before going further, throughout this note, the parameters and functions introduced above are assumed to satisfy the following set on hypothesis.

### Assumption 1.1

We assume that:

i) The constants $\Lambda$, *D*, *b*, $\gamma _1$, $\gamma _2$ and $\mu _h$ are positive.
ii) The functions *p*, $\sigma$ and $\mu _m$ are nonnegative, continuous and 1-periodic.
iii) The function *J* is nonnegative, symmetric, bounded, integrable and $\int _{\mathbb {R}} J(x)\textrm{d}x=1.$ Moreover there is a constant $c>0$ such that $\text {ess}\inf _{x\in (-1,1)}J(x)\ge c$

Now, to analyze (1.1)–(1.2), let us observe that adding up the two equations (1.2) yields the following single equation for $N_m=N_m(t,x)$

1.4 $$\begin{aligned} \partial _t N_m(t,x)= D\partial _{xx}N_m(t,x) + N_m(t,x)\left( b - \mu _m(x) - \int _0^1 K_\epsilon (x-y) N_m(t,y) \, dy\right) ,\nonumber \\ \end{aligned}$$

which describes the evolutionary dynamics of the total density of the mosquito population. This important equation is independent from the rest of the system. Recall that such a problem has been the subject of numerous studies (Ghosh et al. 2024; Génieys et al. 2006; Génieys and Perthame 2007; Genieys et al. 2006; Berestycki and Rossi 2008), particularly to investigate the emergence and reemergence of human epidemics linked to genetic mutations in animals. It is a generalization of the classical Fisher equation, corresponding here to the case where $\epsilon =0$. It accounts for both adaptation and selection within the mosquito population. Note that the selection process is composed by the mortality rate $\mu _m(x)$ and a competition term between mosquitoes which depend explicitly on the biting time *x*. The intraspecific competition among mosquitoes is characterized by their shared dependence on a limited resource: hosts for blood meals. Consequently, the selection results in the density-dependent mortality given by

$$\begin{aligned} \mu _m(x)+\int _0^1 K_\epsilon (x-y) N_m(t,y) \, dy. \end{aligned}$$

The term $\small \int _0^1 K_\epsilon (x-y) N_m(t, y) \, dy$ accounts for mortality induced by competition. For example, if the kernel is set as $K_\epsilon (x) = \frac{1}{2\epsilon } \textbf{1}_{]-\epsilon , \epsilon [}(x)$ then it models a competition, where mosquitoes biting at time *x* are affected by the density of others biting within a temporal window $[x - \epsilon , x + \epsilon ]$. This captures the indirect effects of competition, such as host self-defense triggered by previous bites or anticipatory responses to multiple mosquitoes biting in quick succession. It should be noted that when the competition kernel does not depend on the hour of biting, then the function $K_\epsilon$ equals 1 and this problem finds applications in various fields, notably in ecology (Berestycki and Rossi 2008) and adaptive dynamics (Génieys et al. 2006; Génieys and Perthame 2007; Genieys et al. 2006). Intraspecific competition and mutations in the nonlocal Fisher equation can lead to the emergence of new species (Génieys et al. 2006). We refer to Hamel and Ryzhik (2014) for stability and instability property of the homogeneous equilibrium state, which may lead to the appearance of new (stationary) spatial structures. Additionally, we refer to Génieys et al. (2006), Génieys and Perthame (2007), where the authors focus on the nonlocal Fisher equation from the perspective of adaptive dynamics. Such a model has also bee used in Génieys et al. (2006) to illustrate Darwin’s principle of divergence. In other words, they show how competition in an environment with limited resources can lead to evolutionary branching points. It is important to note that this nonlocal Fisher equation can, in some cases, exhibit Turing instabilities that can be connected to adaptive evolution. A nonlinear analysis of these Turing patterns was proposed in Génieys and Perthame (2007).

In this work, we are interested in studying the evolutionary dynamics of malaria infection in humans and mosquitoes, modeled by (1.1)–(1.2). To that aim, we shall first focus on the analysis of (1.4) which models the total density of mosquitoes structured by their biting hour. We shall more specifically derive a boundedness result for such a problem, by extending some ideas developed in Hamel and Ryzhik (2014). Next, we study the existence and stability of trivial and positive steady-state solutions of (1.4). We focus in particular to the case where $\epsilon \ll 1$, that corresponds to a small frame for the intraspecific competition. Within such a framework, by extending some perturbation arguments, developed in Smith and Waltman (1999), Magal (2009) and the references cited therein, to none-dissipative semiflow we roughly prove some stability property for the positive stationary state of (1.4) when $\epsilon \ll 1$. Finally, based upon this analysis, we investigate the dynamics of the disease modeled by (1.1)–(1.2), particularly the extinction and uniform persistence of the disease.

This note is organized as follows.

In Sect. 2 we state our main results related to (1.4). In Sect. 3, we state and prove basic properties of the full system (1.1)–(1.2). We also state our main results about the uniform persistence of the disease for this system of equations. Numerical simulations and discussions are given in Sects. 4 and 5, respectively. Section 6 is devoted to the derivation of a suitable upper bound for the solutions of (1.4), which is both uniform with respect to time and to $\epsilon$ small enough. We also describe the global dynamics of this problem for $\epsilon \ll 1$. Finally Sect. 7 is concerned with the proof of the uniform persistence results stated in Sect. 3.

## On the mosquito’s population dynamics

Understanding the persistence of mosquitoes is essential for studying the endemicity or eradication of malaria. This section will thus investigate the necessary and sufficient conditions for the long-term persistence or extinction of mosquito populations. Our analysis will focus on the reproductive rate of mosquitoes as a key criterion in determining these dynamics. Specifically, this section is dedicated to studying the equation governing the total mosquito population:

2.1 $$\begin{aligned} {\left\{ \begin{array}{ll} \partial _tN_m(t,x)=D\partial _{xx} N_m(t,x)+N_m(t,x)\left[ b-\mu _m(x)-\int _0^{1} K_\epsilon (x-y)N_m(t,y)\textrm{d}y\right] ,\\ \ x \in (0,1)\\ N_m(t,0)=N_m(t,1)\\ \partial _x N_m(t,0)=\partial _x N_m(t,1)\\ N_m(0,\cdot )=N_{m0}\in C_{per}([0,1];\mathbb {R}) \end{array}\right. } \end{aligned}$$

where $C_{per}([0,1];\mathbb {R})$ denotes the space of functions $\varphi : \mathbb {R} \rightarrow \mathbb {R}$ that are continuous and 1-periodic.

### Existence of nonnegative solution and uniform boundedness

In this section, we investigate the existence of a globally defined nonnegative semiflow for (2.1). Additionally, we establish uniform boundedness, in time, of the semiflow on bounded sets. It is noteworthy that achieving such a uniform boundedness result presents challenges due to the absence of comparison principles for (2.1). To do this, we recast the problem into the framework of an abstract Cauchy problem to use the well-established semigroup theory. Let $X=C_{per}([0,1];\mathbb {R})$ denote the space of functions $\varphi : \mathbb {R} \rightarrow \mathbb {R}$ that are continuous and 1-periodic. Let $X_+=C_{per}([0,1];\mathbb {R}_+)$ denote the positive cone of *X* with partial order

$$\begin{aligned} \varphi \le \psi \Longleftrightarrow \psi -\varphi \in X_+. \end{aligned}$$

Subsequently, the Banach space *X* is equipped with the standard supremum norm defined as

$$\begin{aligned} \Vert u\Vert :=\sup _{x\in [0,1]}\vert u(x)\vert ,\ \forall u \in X. \end{aligned}$$

Next, we consider the diffusion operator $A: D(A)\subseteq X \rightarrow X$, defined as

2.2 $$\begin{aligned} Au=Du'',\ \forall u \in D(A):=\lbrace u\in X: \, u''\in X\rbrace . \end{aligned}$$

Additionally, we introduce the nonlinear operator $F_\epsilon : X \rightarrow X$, defined for all $\epsilon \in [0,\epsilon _0]$, by

2.3 $$\begin{aligned} F_\epsilon (u):=bu-\mu _m u-u\int _0^{1}K_\epsilon (\cdot -y)u(y)\textrm{d}y,\, \forall u \in X. \end{aligned}$$

Finally, setting $u(t):=N_m(t,\cdot )$ for all $t>0$ and $u_0:=N_m(0,\cdot )$ the system (2.1) can be put into the following abstract form:

2.4 $$\begin{aligned} {\left\{ \begin{array}{ll} \dfrac{\textrm{d}u(t)}{\textrm{d}t}=Au(t)+F_\epsilon (u(t)), \ \forall t>0\\ u(0)=u_0 \in X_+. \end{array}\right. } \end{aligned}$$

The linear operator *A* defined in (2.2) corresponds to the Laplace operator with periodic boundary conditions. As a consequence, it generates a positive analytic semigroup $\{T_{A}(t)\}_{t\ge 0} \subset \mathcal {L}(X)$, which, for each $u_0 \in X$ and $t>0$, is expressed as

2.5 $$\begin{aligned} (T_{A}(t)u_0)(x)=\dfrac{1}{\sqrt{4\pi Dt}}\sum _{k\in \mathbb {Z}}\int _0^1 e^{-\frac{(x-y-k)^2}{4Dt}}u_0(y)\textrm{d}y, \ \forall x \in [0,1]. \end{aligned}$$

Moreover, the semigroup $\{T_{A}(t)\}_{t\ge 0} \subset \mathcal {L}(X)$ is a contraction semigroup on *X*, satisfying

2.6 $$\begin{aligned} \Vert T_{A}(t) u_0\Vert \le \Vert u_0\Vert ,\ \forall u_0 \in X,\ \forall t\ge 0. \end{aligned}$$

The first main result of this section is the following.

#### Proposition 2.1

Let Assumption 1.1 be satisfied. Let $\epsilon \ge 0$ be given and fixed. Then (1.1) generates a globally defined nonnegative semiflow $\{U_\epsilon (t,\cdot )\}_{t\ge 0}$ on $X_+$. More precisely, for each $u_0 \in X_+$, the map defined as $u(t):=U_\epsilon (t,u_0)$ for all $t\ge 0$ is the unique nonnegative mild solution to (1.1) with initial condition $u_0$ at time $t=0$, and

2.7 $$\begin{aligned} \Vert U_\epsilon (t;u_0)\Vert \le e^{bt } \Vert u_0 \Vert ,\ \forall t\ge 0. \end{aligned}$$

Furthermore, for each $u_0 \in X_+$, the function $U_\epsilon (\cdot ,u_0)$ is of class $C^\infty$ on $(0,+\infty )\times [0,1]$.

#### Proof

It can be readily verified that the nonlinear map $F_\epsilon$ defined in (2.3) is Lipschitz continuous on bounded sets of $X_+$. Additionally, for any bounded set $B:=\{ u_0 \in X_+: \Vert u_0 \Vert \le \eta \}$, with $\eta >0$, there exists $\lambda :=\lambda (B)>0$ such that $\lambda u_0 +F_\epsilon (u_0)\in X_+$ for all $u_0\in B$. Thanks to Magal et al. (2018), for each $u_0\in X_+$ there exists a maximally defined solution to (1.1). More precisely, there exists $\tau :=\tau (u_0)\in (0,+\infty ]$ such that

$$\begin{aligned} u(t)=T_{A}(t)u_0+\int _0^t T_{A}(t-s)F_\epsilon (u(s)) \textrm{d}s,\ \forall t \in [0,\tau ). \end{aligned}$$

Next, recalling the definition of $F_\epsilon : X \rightarrow X$ in (2.3), it comes

$$\begin{aligned} u(t)\le T_{A}(t)u_0+\int _0^t b T_{A}(t-s)u(s) \textrm{d}s,\ \forall t \in [0,\tau ) \end{aligned}$$

from where Magal et al. (2019), $u(t)\le v(t)$ for all $t \in [0,\tau )$ with

2.8 $$\begin{aligned} v(t)= &  T_{A}(t)u_0+b\int _0^t T_{A}(t-s)v(s) \textrm{d}s,\ \forall t \in [0,\tau ) \Longleftrightarrow v(t)=e^{bt} T_{A}(t)u_0, \nonumber \\  &  \qquad \ \forall t\in [0,\tau ). \end{aligned}$$

Since the norm of *X* is monotone in $X_+$, we deduce from (2.8) and the inequality between *u* and *v* on $[0,\tau )$ that

2.9 $$\begin{aligned} \Vert u(t) \Vert \le e^{bt } \Vert u_0 \Vert ,\ \forall t\in [0,\tau ). \end{aligned}$$

We conclude from (2.9) that the solutions exist globally defined in time that is $\tau :=\tau (u_0)=+\infty$ for all $u_0 \in X_+$. To conclude the proof, we recall that the semigroup generated by the Laplace operator *A* is analytic on $(0,+\infty )$ and $T_{A}(t)u_0 \in D(A)$ for each $t>0$. The proof is now completed since $F_\epsilon$ is of class $C^\infty$ on *X*. $\square$

The second main result of this section concerns the uniform boundedness in time of the semiflow. It is expressed as follows.

#### Theorem 2.2

Let Assumption 1.1 be satisfied. Then for any initial condition $u_0\in X_+$, the solution of (2.1) is globally bounded on $[0,\infty )\times \mathbb {R}\times [0,\epsilon _0]$, that is for all $t\ge 0$, $x\in \mathbb {R}$ and $\epsilon \in [0,\epsilon _0]$,

$$\begin{aligned} 0\le U_\epsilon (t;u_0)(x)\le e^b\max \left( \Vert u_0\Vert ,\frac{b}{c}\right) \left( 1+\frac{\epsilon _0}{\sqrt{\pi D}}\right) . \end{aligned}$$

The proof of Theorem 2.2 is given in Sect. 6.

### Extinction and persistence of the mosquitoes

To characterize the extinction or persistence of the mosquito population in terms of the mosquitoes’ reproductive rate, we make the assumption that their distribution is close to the equilibrium state $\bar{u} \equiv 0$. This allows us to locally approximate the dynamics with the following system:

2.10 $$\begin{aligned} {\left\{ \begin{array}{ll} \partial _t w(t,x) = D\partial _{xx}w(t,x) - \mu _m(x)w(t,x) + bw(t,x), & t > 0, x \in \mathbb {R}\\ w(0,\cdot ) = w_0 \in X. \end{array}\right. } \end{aligned}$$

Let $L: D(A)\subset X \rightarrow X$ be the linear operator encountering the adaptation process of the biting time and the mortality, that is,

2.11 $$\begin{aligned} L\phi :=A\phi -\mu _m \phi , \ \forall \phi \in D(A), \end{aligned}$$

where $A: D(A) \subset X \rightarrow X$ is defined in (2.2). Therefore, the semigroup $\{T_{L}(t)\}_{t\ge 0}$, generated by *L*, represents the survival operator. Let $w_0\in X_+$ be an initial distribution of the population at time $t=0$. Solving (2.10) by the variation of constants formula and applying the birth operator $\phi \mapsto \mathcal {K}\phi :=b \phi$ we obtain

$$\begin{aligned} \mathcal {K}w(t,\cdot )= b T_{L}(t)w_0+\mathcal {K} \int _0^t T_{L}(t-s) \mathcal {K}w(s,\cdot ) \textrm{d}s,\ \forall t\ge 0. \end{aligned}$$

Since the spectral bound of *L* is negative, one can follow the methodology outlined in Inaba (2012), to define the next-generation operator

$$\begin{aligned} w_0 \in X \mapsto \mathcal {G}w_0:= \mathcal {K} \int _0^\infty T_{L}(t)w_0 \textrm{d}t=b (-L)^{-1}w_0. \end{aligned}$$

This operator encapsulates the effects of mosquito reproduction, survival, and population growth over time. Therefore, we can define the reproductive rate of mosquitoes as the spectral radius of the next-generation operator:

2.12 $$\begin{aligned} \mathcal {T}_0:=\textsf{r}(\mathcal {G})=\textsf{r}(\mathcal {K} (-L)^{-1})=b \ \textsf{r}((-L)^{-1}). \end{aligned}$$

The result concerning mosquito extinction is as follows.

#### Theorem 2.3

(Extinction) Let Assumption 1.1 be satisfied. If $\mathcal {T}_0\le 1$ then for each initial distribution $u_0 \in X_+$ we have $U_\epsilon (t,u_0) \rightarrow 0$ when $t\rightarrow +\infty$.

The proof of Theorem 2.3 is postponed in Sect. 7. Let us now state the main result concerning the persistence of the mosquito population.

#### Theorem 2.4

(Persistence of stability) Let Assumption 1.1 be satisfied. If $\mathcal {T}_0>1$ then there exists $\xi _0\in [0,\epsilon _0]$ and a continuous map $\varphi :[0,\xi _0]\rightarrow X_+$ such that for each $\epsilon \in [0,\xi _0]$, $\varphi (\epsilon )$ is an equilibrium state of (2.1) with $\varphi (\epsilon ) \in \text {int}(X_+)$. Additionally, for any bounded set $B\subset X_+\setminus \{0\}$, there exists $\xi _1\in [0,\xi _0]$ such that for all $\epsilon \in [0,\xi _1]$ and $\psi \in B$, we have

$$\begin{aligned} U_\epsilon (t;\psi )\rightarrow \varphi (\epsilon ),\ \text {as}\ t\rightarrow \infty . \end{aligned}$$

Furthermore, if $\epsilon =0$ then the equilibrium state $\varphi _0:=\varphi (0)\in \text {int}(X_+)$ is globally asymptotically stable in $X_+$.

The above result shows that the global stability of the equilibrium when $\epsilon =0$ can be perturbed for small values of $\epsilon$. However, when $\epsilon$ is small, the persistent equilibrium is not necessarily globally stable and its domain of attraction may depend on $\epsilon$.

## On the disease dynamics

Faced with the physiological and behavioral changes in mosquitoes observed in recent decades, understanding the evolutionary dynamics of these insects becomes crucial. In this section, we focus on the evolutionary dynamics of malaria, taking into account mosquito adaptation and competition.

### Existence and uniqueness of nonnegative solution

In this subsection, we investigate the existence and uniqueness of nonnegative solutions of (1.1)–(1.2). To this end, we rewrite the system as an abstract Cauchy problem in a suitable Banach space. Consider the space $Y=\mathbb {R}\times \mathbb {R}\times X\times X,$ with its associated positive cone $Y_+=\mathbb {R}_+\times \mathbb {R}_+\times X_+\times X_+$. Let us define the subset $\mathcal {X}$ as follows $\mathcal {X}=Z\times X_+\times X_+,$ where

$$\begin{aligned} Z:=\{(S_h, I_h)\in \mathbb {R}_+\times \mathbb {R}_+: S_h+I_h\ge \delta \} \end{aligned}$$

and $\delta$ is a positive constant belonging to $(0,\Lambda /(\gamma _2+\mu _h))$. To apply the semigroup theory (Pazy 2012), we define the operator (*H*, *D*(*H*)) on $\mathcal {X}$ by $D(H)=\mathbb {R}\times \mathbb {R}\times D(A)\times D(A)$ and

3.1 $$\begin{aligned} H\begin{pmatrix} u_1\\ u_2\\ v_1\\ v_2 \end{pmatrix}:=\begin{pmatrix} 0\\ 0\\ D\Delta v_1\\ D\Delta v_2 \end{pmatrix}, \forall (u_1,u_2,v_1,v_2)\in D(H). \end{aligned}$$

Then, *H* generates an analytic, and compact semigroup $\{T_H(t)\}_{t\ge 0}$ on $Y_+$ given for any $(u_1,u_2,v_1,v_2)\in Y_+$ by

3.2 $$\begin{aligned} T_H(t)\begin{pmatrix} u_1\\ u_2\\ v_1\\ v_2 \end{pmatrix}=\begin{pmatrix} u_1\\ u_2\\ T_A(t)v_1\\ T_A(t)v_2 \end{pmatrix},\ \forall t\ge 0. \end{aligned}$$

Next, we introduce the nonlinear part of (1.1)–(1.2), the map $G_\epsilon :\mathcal {X}\rightarrow Y_+$ as follows:

3.3 $$\begin{aligned} G_\epsilon (U) &  :=\begin{pmatrix} \Lambda -\frac{u_1}{u_1+u_2}\int _0^1p(y)v_2(y)\textrm{d}y+\gamma _1u_2-\mu _hu_1\\ \quad \\ \frac{u_1}{u_1+u_2}\int _0^1p(y)v_2(y)\textrm{d}y-(\mu _h+\gamma _1+\gamma _2)u_2\\ \quad \\ -\sigma \frac{u_2}{u_1+u_2}v_1+b(v_1+v_2)-v_1\left( \mu _m+K_\epsilon *(v_1+v_2)\right) \\ \quad \\ \sigma \frac{u_2}{u_1+u_2}v_1-v_2\left( \mu _m+K_\epsilon *(v_1+v_2)\right) \end{pmatrix},\ \nonumber \\ \forall U &  :=\begin{pmatrix} u_1\\ u_2\\ v_1\\ v_2 \end{pmatrix}\in \mathcal {X},\ \forall \epsilon \in [0,\epsilon _0]. \end{aligned}$$

Hence, setting $u(t):=(S_h(t),I_h(t), S_m(t,\cdot ), I_m(t,\cdot ))$, the system (1.1)–(1.2) can be rewritten as follows

$$\begin{aligned} {\left\{ \begin{array}{ll} \dfrac{\textrm{d}u(t)}{\textrm{d}t}=Hu(t)+G_\epsilon (u(t)),\ \forall t>0,\, \epsilon \in [0,\epsilon _0],\\ u(0)=u_0\in \mathcal {X}, \end{array}\right. } \end{aligned}$$

with $$u_0=(S_{0h}, I_{0h}, S_{0m}, I_{0m})\in \mathcal {X}$$.

#### Proposition 3.1

Let Assumption 1.1 be satisfied. Let $\epsilon \in [0,\epsilon _0]$ be given and fixed. Then, (1.1)–(1.2) has a unique globally defined mild solution $\Phi _\epsilon (t,\cdot )\in C([0,+\infty );\mathcal {X})$. Specifically, for each initial condition $v_0:=(S_{0h}, I_{0h}, S_{0m}, I_{0m})\in \mathcal {X}$, the map $t\mapsto \Phi _\epsilon (t,v_0)$ is a unique nonnegative mild solution to (1.1)–(1.2).

#### Proof

To prove the existence of solutions, we will use the results from Magal et al. (2019), Thieme (1990), Dieye et al. (2022). First, the operator *H* generates a compact and analytic semigroup. To establish the existence of a solution to (1.1)–(1.2), it is sufficient to show that the nonlinear part of (1.1)–(1.2), given in (3.3), is Lipschitz continuous and that the conditions of the Nagumo–Brezis theorem (Thieme 1990) are satisfied. Additionally, it can be easily verified that the function $G_\epsilon$ is Lipschitz continuous on bounded sets of $\mathcal {X}$. To complete the proof, we must show that (1.1)–(1.2) satisfies the subtangential condition. Let $\lambda >0$ be given and sufficiently large. Then, the resolvent of *H* satisfies

3.4 $$\begin{aligned} \lambda (\lambda \textrm{I}_{Y}-H)^{-1}u=\begin{pmatrix} u_1\\ u_2\\ \lambda (\lambda \textrm{I}_{X}-A)^{-1}v_1\\ \lambda (\lambda \textrm{I}_{X}-A)^{-1}v_1 \end{pmatrix},\ \forall u:=\begin{pmatrix} u_1\\ u_2 \\ v_1 \\ v_2 \end{pmatrix}\in \mathcal {X}. \end{aligned}$$

Since the operator *A* is positive resolvent, we infer from (3.4) that $\lambda (\lambda \textrm{I}_Y -H)^{-1}\mathcal {X}\subset \mathcal {X}$. Finally, we will show that for each $\xi >0$, we have

3.5 $$\begin{aligned} u+tG(u)\in \mathcal {X},\, t> 0, \end{aligned}$$

for $t>0$ small enough, $u\in \mathcal {X}$, $\Vert u\Vert _Y\le \eta$. Let $\eta >0$ be given and fixed. Let us define

$$\begin{aligned} m_0:=\min \left( 1,\frac{1}{\mu _h+\gamma _1+\gamma _2},\frac{\delta }{\mu _h\delta +\eta \Vert p\Vert }, \frac{1}{\eta +\Vert \sigma \Vert +\Vert \mu _m\Vert }, \frac{1}{\eta \Vert p\Vert }\right) . \end{aligned}$$

Then, for any $0<t\le m_0$, and $u:=(u_1,u_2,v_1,v_2)\in \mathcal {X}$ such that $\Vert u\Vert _Y\le \eta$, we have

$$\begin{aligned} \begin{pmatrix} u_1\\ u_2\\ v_1\\ v_2 \end{pmatrix}+tG\begin{pmatrix} u_1\\ u_2\\ v_1\\ v_2 \end{pmatrix}=\begin{pmatrix} V_1(t,u_1,u_2,v_1,v_2)\\ V_2(t,u_1,u_2,v_1,v_2)\\ V_3(t,u_1,u_2,v_1,v_2)\\ V_4(t,u_1,u_2,v_1,v_2) \end{pmatrix}. \end{aligned}$$

where we have set

$$\begin{aligned} {\left\{ \begin{array}{ll} V_1(t,u_1,u_2,v_1,v_2)= u_1+t\left[ \Lambda -\dfrac{u_1}{u_1+u_2}\int _0^1p(y)v_2(y)\textrm{d}y+\gamma _1u_2-\mu _hu_1\right] \quad \\ \,\\ V_2(t,u_1,u_2,v_1,v_2)=u_2+t\left[ \dfrac{u_1}{u_1+u_2}\int _0^1p(y)v_2(y)\textrm{d}y-(\mu _h+\gamma _1+\gamma _2)u_2\right] \\ \,\\ V_3(t,u_1,u_2,v_1,v_2)=v_1(1+t[-\sigma \dfrac{u_2}{u_1+u_2}-\left( \mu _m+K_\epsilon *(v_1+v_2)\right) ])+tb(v_1+v_2)\\ \,\\ V_4(t,u_1,u_2,v_1,v_2)=v_2(1-t\left( \mu _m+K_\epsilon *(v_1+v_2)\right) )+t\sigma \dfrac{u_2}{u_1+u_2}v_1. \end{array}\right. } \end{aligned}$$

Our aim is to show that for any $t\in (0,m_0)$, and $u\in B_Y(0,\eta )$, we have

$$\begin{aligned} V_1(t,u)\ge 0,\ V_2(t,u)\ge 0,\, V_1(t,u)+V_2(t,u)\ge \delta ,\, V_3(t,u)\ge 0\ \text { and }\, V_4(t,u)\ge 0. \end{aligned}$$

Since $t\in (0,m_0)$, it is readily verified that for every $u\in B_Y(0,\eta )$, we have

$$\begin{aligned} V_1(t,u)\ge 0,\ V_2(t,u)\ge 0,\ V_3(t,u)\ge 0\, \text { and } V_4(t,u)\ge 0. \end{aligned}$$

It remains to prove that for every $u\in B_Y(0,\eta )$ and $t\in (0,m_0)$, we have $V_1(t,u)+V_2(t,u)\ge \delta$. To this end, we observe that for all $u\in B_Y(0,\eta )$ and $t\in (0,m_0)$, we have

3.6 $$\begin{aligned} \begin{aligned} V_1(t,u)+V_2(t,u)&= u_1+t(\Lambda -\mu _hu_1)+u_2-t(\mu _h+\gamma _2)u_2\\&=u_1+u_2+t\Lambda -t(\mu _h+\gamma _2)(u_1+u_2)+\gamma _2u_1\\&\ge u_1+u_2+t\Lambda -t(\mu _h+\gamma _2)(u_1+u_2) \end{aligned} \end{aligned}$$

and it follows from (3.6) that for every $u\in B_Y(0,\eta )$ and $t\in (0,m_0)$, we have $V_1(t,u)+V_2(t,u)\ge \delta$. Consequently, (3.5) holds.

Thus, using (3.4) and (3.5), we infer from Dieye et al. (2022) that (1.1)–(1.2) has maximally defined mild solution. Additionally, for all $t\ge 0$, we have

$$\begin{aligned} {\left\{ \begin{array}{ll} S_h(t) + I_h(t) \le \max \left( S_{0h}+I_{0h}, \frac{\Lambda }{\mu _h}\right) \\ \Vert S_m(t,\cdot )\Vert + \Vert I_m(t,\cdot ) \Vert \le e^{bt}(\Vert S_{0m}\Vert +\Vert I_{0m}\Vert ). \end{array}\right. } \end{aligned}$$

Therefore, the solution generated by (1.1)–(1.2) is global. $\square$

### Extinction and persistence of the disease

This section focuses on the main results regarding the full model (1.1) and (1.2). Thanks to Theorem 2.3, if $\mathcal {T}_0\le 1$, then the mosquito population undergoes extinction. Therefore, using the human’s equation it is readily proved that $I_h(t) \rightarrow 0$ when $t \rightarrow +\infty$ while the susceptible individuals stabilize at $\bar{S}_h:=\Lambda /\mu$. Consequently, it is natural to consider, in the following, the invasion process whenever $\mathcal {T}_0>1$ and the populations of humans and mosquitoes are in a disease-free state $\bar{E}(\epsilon ):=(\bar{S}_h,\bar{I}_h,\bar{S}_m(\epsilon ),\bar{I}_m)$ with

3.7 $$\begin{aligned} \bar{S}_h:=\frac{\Lambda }{\mu },\ \bar{I}_h=0,\ \bar{S}_m(\epsilon ):=\varphi (\epsilon ),\ \text { and } \bar{I}_m\equiv 0 \end{aligned}$$

where $\varphi (\epsilon )$, $\epsilon \in [0,\xi _1]$ is the equilibrium state of the mosquito given in Theorem 2.4. To investigate disease invasion dynamics within the human and mosquito populations, we examine the linearized equation of the infective compartment of (1.1)–(1.2) at $\bar{E}$. This is expressed as:

$$\begin{aligned} {\left\{ \begin{array}{ll} \dfrac{\textrm{d}I_h(t)}{\textrm{d}t}=\int _0^{1} p(y)I_m(t,y)\textrm{d}y -(\gamma _1+\gamma _2)I_h(t)-\mu _hI_h(t), \\ \dfrac{\partial I_m(t,x)}{\partial t}=D\partial _{xx} I_m(t,x)+\sigma (x)\dfrac{\bar{S}_m^\epsilon (x)}{\bar{S}_h} I_h(t)-(\mu _m(x)+q_\epsilon (x)) I_m(t,x)\\ I_h(0)=I_h\in \mathbb {R},\ I_m(0,\cdot )=I_{m0}\in X \end{array}\right. } \end{aligned}$$

with

$$\begin{aligned} q_\epsilon (x):=\int _0^1 K_\epsilon (x-y)\bar{S}_m^\epsilon (y) \textrm{d}y,\ \forall x \in \mathbb {R}. \end{aligned}$$

To define the basic reproduction number, we introduce two linear operators: $\mathcal {F}: \mathbb {R}\times X \rightarrow \mathbb {R}\times X$ representing new infections, and $\mathcal {V}: \mathbb {R}\times D(A) \rightarrow \mathbb {R}\times X$ (*D*(*A*) is domain of the Laplace operator defined in (2.3)) representing other processes such as adaptations, death, and recovery. They are defined as follows:

$$\begin{aligned} \mathcal {F}\begin{pmatrix} I_h\\ I_m \end{pmatrix}:=\begin{pmatrix} \int _0^{1} p(y)I_m(y)\textrm{d}y\\ \sigma \frac{\bar{S}_m^\epsilon (\cdot )}{\bar{S}_h} I_h \end{pmatrix},\quad \forall \begin{pmatrix} I_h\\ I_m \end{pmatrix} \in \mathbb {R}\times X \end{aligned}$$

and $\mathcal {V}: \mathbb {R}\times D(A) \rightarrow \mathbb {R}\times X$ the linear operator

$$\begin{aligned} \mathcal {V}\begin{pmatrix} I_h\\ I_m \end{pmatrix}:=\begin{pmatrix} -(\gamma _1+\gamma _2+\mu _h) I_h\\ L_\epsilon I_m \end{pmatrix},\ \forall \begin{pmatrix} I_h\\ I_m \end{pmatrix}\in \mathbb {R}\times D(A), \end{aligned}$$

where

3.8 $$\begin{aligned} L_\epsilon I_m:=D\partial _{xx} I_m-(\mu _m+q_\epsilon ) I_m,\ \forall I_m \in D(A). \end{aligned}$$

Therefore, we can define the basic reproduction number as the spectral radius of $\mathcal {F}(-\mathcal {V})^{-1}$ that is

$$\begin{aligned} \mathcal {R}_{0,\epsilon }:=\textsf{r}(\mathcal {F}(-\mathcal {V})^{-1}). \end{aligned}$$

Let us now note that the linear operator $\mathcal {F}(-\mathcal {V})^{-1}$ is given by

$$\begin{aligned} \mathcal {F}(-\mathcal {V})^{-1}\begin{pmatrix} I_h\\ I_m \end{pmatrix}=\begin{pmatrix} \int _0^{1} p(y)(-L_\epsilon )^{-1}I_m(y) dy\\ \dfrac{\sigma (x)}{\gamma _1+\gamma _2+\mu _h}\frac{\bar{S}_m^\epsilon (\cdot )}{\bar{S}_h} I_h \end{pmatrix},\quad \forall \begin{pmatrix} I_h\\ I_m \end{pmatrix} \end{aligned}$$

from where using similar arguments in [Lemma 2.3, Djidjou-Demasse et al. 2022] together with the fact that

$$\begin{aligned} (-L_\epsilon )^{-1}=\int _0^\infty T_{L_\epsilon }(t) \textrm{d}t \end{aligned}$$

we obtain that the basic reproduction number $\mathcal {R}_{0,\epsilon }$ is given by

3.9 $$\begin{aligned} \mathcal {R}_{0,\epsilon }:=\dfrac{1}{\gamma _1+\gamma _2+\mu _h} \int _0^{1} p(y) \left( \int _0^\infty T_{L_\epsilon }(t)\frac{\bar{S}_m^\epsilon (\cdot )}{\bar{S}_h}\sigma \textrm{d}t\right) (y) dy. \end{aligned}$$

The main result of this section is the following.

#### Theorem 3.2

Let Assumption 1.1 be satisfied, and assume that $$\mu _1>0$$. If $$\mathcal {R}_{0,\epsilon }>1$$ then for all bounded subset $$\Omega $$ of $$Y_+$$, there exists $$\zeta ^{**}:=\zeta ^{**}(\Omega )>0$$ such that for every $$(S_{0h},I_{0h},S_{0m},I_{0m})\in \Omega {\setminus }\partial \mathcal {M}_\Omega $$ with $$N_{0m}\not \equiv 0$$, we have

$$\begin{aligned} \liminf _{t\rightarrow +\infty } (I_h(t)+\Vert I_m(t,\cdot )\Vert )\ge \zeta ^{**}, \end{aligned}$$

where $(S_h,I_h,S_m,I_m)$ denotes the solution of (1.1) with the initial data $(S_{0h},I_{0h},S_{0m},I_{0m})$ and $\partial \mathcal {M}_\Omega$ is the set defined in (8.2).

## Numerical simulations

Malaria is a potentially life-threatening infectious disease transmitted to humans through the bites of infected mosquitoes of the *Anopheles* genus. It represents a major public health issue, particularly in tropical and subtropical regions. Over the past decades, control interventions have primarily targeted the nocturnal activity of mosquitoes. These measures-most notably insecticide-treated bed nets (ITNs) and indoor residual spraying (IRS)-have significantly reduced malaria-related mortality in many regions of Africa, with marked benefits for children (Gatton et al. 2013; O’Meara et al. 2010; Ndenga et al. 2016).

However, as reported in Carrasco et al. (2019), Moiroux et al. (2012), Sangbakembi-Ngounou et al. (2022), Yohannes and Boelee (2012), extensive use of these interventions may induce behavioral adaptations in mosquito populations. Such adaptive responses could mitigate the efficacy of current control measures and foster residual transmission. In this context, we use a mathematical model to study the adaptive behavior of mosquitoes, particularly focusing on the daily rhythm of their biting activity.

**Fig. 1:**
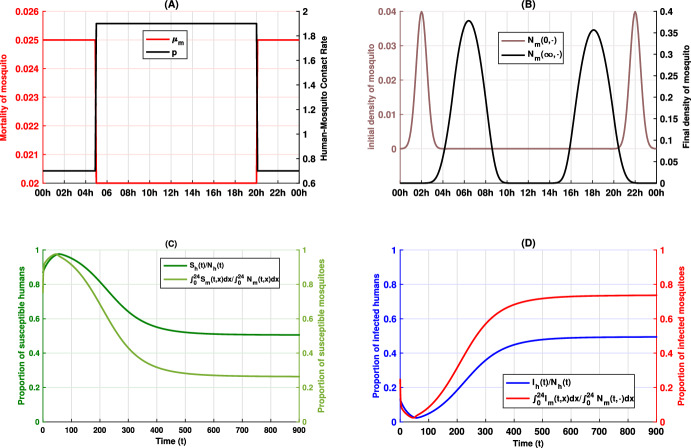
Dynamics of the epidemic and shifts in mosquito activity patterns for scenario 1. Panel **A** illustrates the human-mosquito contact rate $x \mapsto p(x)$ alongside the mortality rate $x \mapsto \mu _m(x)$. Panel **B** compares the initial and final mosquito activity distributions, while Panels **C** and **D** depict the temporal evolution of the epidemic in the human and mosquito populations, respectively

**Fig. 2:**
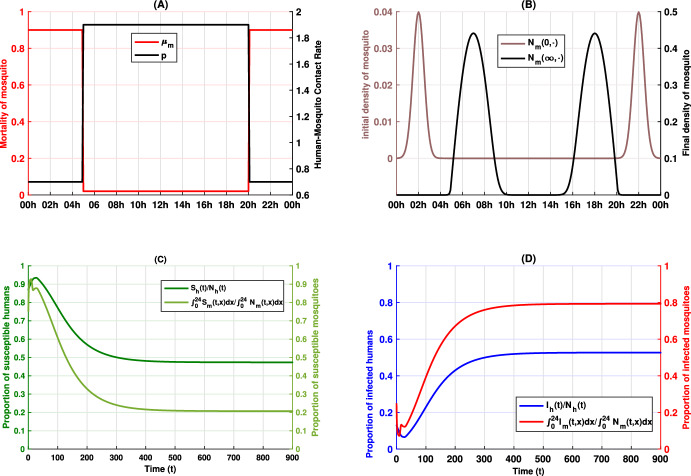
Dynamics of the epidemic and shifts in mosquito activity patterns for scenario 2. Panel **A** illustrates the human-mosquito contact rate $x \mapsto p(x)$ alongside the mortality rate $x \mapsto \mu _m(x)$. Panel **B** compares the initial and final mosquito activity distributions, while Panels **C** and **D** depict the temporal evolution of the epidemic in the human and mosquito populations, respectively

**Fig. 3:**
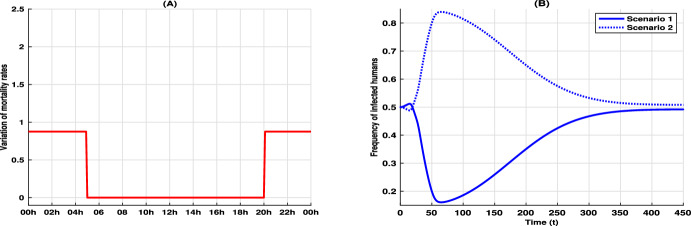
Epidemiological implications of mosquito adaptations. Panel **A** shows the increase in mosquito mortality from scenario 2 to scenario 1 i.e. $x\mapsto \mu _{m,2}(x)-\mu _{m,1}(x)$, with $x \mapsto \mu _{m,i}(x)$ the mortality rate for scenario *i*. Panel **B** displays the ratio of the number of infected humans in scenario 1 (resp. Scenario 2) to the combined number of infected humans across both scenarios i.e. $t\mapsto I_{h,i}(t)/(I_{h,1}(t)+I_{h,2}(t))$ with $I_{h,i}(t)$ the number of infected humans for scenario *i*

We conducted numerical simulations under two scenarios based on our model, detailed in Equations (1.1), (1.2), and (2.1), to highlight the temporal dynamics of biting behavior and its influence on disease transmission. In our simulations, the biting period is represented over the full 24-hour cycle (00:00–24:00) rather than the normalized interval [0,1]. In the two scenarios, all parameters remain fixed except for the mosquito mortality rate $\mu _m$ which will be used to encode the control measures. Figures 1A and 2A illustrate the mortality rates $\mu _m(x)$ for each scenario, while Fig. 3A displays the differences between these rates. In our simulations, a higher mortality rate is imposed during peak mosquito activity periods (20:00–00:00 and 00:00–05:00), with the rate remaining unchanged during the assumed lower activity period (05:00–20:00). This modulation reflects the implementation of control measures such as ITNs and IRS. To quantify the impact of control interventions on mosquito behavior and malaria transmission, we define the total human population $N_h(0)=S_h(0)+I_h(0)$ and the total density of mosquito populations $N_m(0,\cdot ) = S_m(0,\cdot )+I_m(0,\cdot )$ at the initial time $t = 0$, with $S_h(0) = 100$ $I_h(0) = 15$, and

4.1 $$\begin{aligned} N_m(0, x) = 10^{-2}\times \dfrac{5\sqrt{2}}{\sqrt{\pi }}\left( e^{-4(x-2)^2}+ e^{-4(x-22)^2}\right) , \forall x\in [0,24] \end{aligned}$$

with the initial susceptible and infected mosquitoes given by

4.2 $$\begin{aligned} S_m(0,x) = 0.75\times N_m(0,x),\, \ \text { and } I_m(0,x) = 0.25\times N_m(0,x), \forall x\in [0,24]. \end{aligned}$$

The mosquito mortality rates $\mu _m(x)$ for two scenario are given by

4.3 $$\begin{aligned} \text {(Scenario 1) }\quad \mu _m(x) ={\left\{ \begin{array}{ll} 0.02, & \text {if } x\in [5,20], \\ 0.025, & \text {if } x\in [0,5[\cup ]20, 24], \end{array}\right. } \end{aligned}$$

and

4.4 $$\begin{aligned} \text {(Scenario 2) }\quad \mu _m(x) = {\left\{ \begin{array}{ll} 0.02, & \text {if } x\in [5,20], \\ 0.9, & \text {if } x\in [0,5[\cup ]20, 24]. \end{array}\right. } \end{aligned}$$

The human-mosquito contact rate *p*(*x*) is defined as

4.5 $$\begin{aligned} p(x) = {\left\{ \begin{array}{ll} 1.9, & \text {if } x\in [5,20], \\ 0.7, & \text {if } x\in [0,5[\cup ]20, 24]. \end{array}\right. } \end{aligned}$$

The other parameters used in the simulations are set as follows: $\Lambda = 1.7$, $\gamma _1 = 0.01$, $\gamma _2 = 0.015$, $\mu _h = 0.01$, $D = 0.005$, $b = 0.9$, $\sigma (x) = 0.9p(x)$, and $\epsilon = 7$. Note that the mortality and contact rates are modeled as step functions for simplicity. However, they can be approximated by continuous functions with the desired precision without affecting the simulation results.

Figures 1B and 2B demonstrate that, following an initial high activity period-where host protection mechanisms enforce a high mortality-the mosquitoes shift their biting activity to periods with lower mortality. This behavioral adaptation is in line with observations reported in Carrasco et al. (2019), Moiroux et al. (2012), Sangbakembi-Ngounou et al. (2022), Yohannes and Boelee (2012), Ndenga et al. (2016) and suggests that the selective pressure from control measures can induce significant changes in mosquito activity patterns. Furthermore, in Fig. 3B we present, for each scenario, the ratio at each time *t* of the total number of infected humans in that scenario to the sum of infected individuals across both scenarios. The results indicate that an increased mortality rate during the same time window may lead to a higher number of infected individuals, thereby emphasizing the epidemiological implications of mosquito adaptation.

## Discussions and conclusion

Our analysis demonstrates that prolonged use of insecticide-based interventions may inadvertently select for behavioral adaptations in mosquito populations, particularly changes in the daily biting rhythm (Carrasco et al. 2019; Moiroux et al. 2012; Sangbakembi-Ngounou et al. 2022; Yohannes and Boelee 2012). Although the model provides valuable insights into the evolutionary dynamics driving these adaptations, several limitations should be noted.

First, the model assumes a mosquito birthrate that is independent of the biting time trait, thus overlooking potential evolutionary trade-offs that could affect population dynamics (Li et al. 2019; Li and Ainseba 2020). Moreover, larval competition is not included in our framework, despite its potential influence on adult mosquito density (Carrasco et al. 2019; Feng and Castillo-Chavez 2006; Paaijmans et al. 2009). The absence of an age-structured human population further limits our ability to capture the heterogeneity in infection dynamics (Qu et al. 2023; Richard et al. 2022). One may also consider genetic difference in susceptible humans as in Feng and Castillo-Chavez (2006), Beck et al. (1984).

Additionally, the simplified representation of mosquito-human interactions does not account for factors such as human movement, individual immunity, and seasonal variations in mosquito behavior, all of which may impact disease transmission (Xu and Zhang 2015; Qu et al. 2023; Chitnis et al. 2012; Djidjou-Demasse et al. 2020; Ewing et al. 2016). Despite these limitations, our model successfully illustrates how selective pressure by insecticide-treated bed nets (ITNs) and indoor residual spraying (IRS) can drive shifts in mosquito biting behavior and alter epidemiological outcomes (Gatton et al. 2013; Sangbakembi-Ngounou et al. 2022).

Future work should focus on refining the model by incorporating data-driven parametrization and explicit evolutionary trade-offs. Expanding the model to include more complex mosquito-human interactions such as seasonality, heterogeneous human populations, and detailed mosquito population dynamics will be essential for improving its applicability to real-world settings and for developing more effective vector control strategies.

In conclusion, our numerical simulations and subsequent analysis underscore the importance of considering behavioral adaptations in mosquitoes when evaluating the long-term impact of vector control measures (Sangbakembi-Ngounou et al. 2022; Ferreira et al. 2017; Gatton et al. 2013; Carrasco et al. 2019). A deeper understanding of these dynamics is critical for the design of robust interventions aimed at reducing the global burden of malaria (Gatton et al. 2013).

## Proof of Theorem 2.2: uniform boundedness

This section is devoted to the proof of Theorem 2.2. Our initial step entails proving the following lemma.

### Lemma 6.1

Let Assumption 1.1 be satisfied. Let $\epsilon >0$ be given and fixed. Then each $u_0\in X_+$, the function $v: [0,+\infty )\times \mathbb {R}\rightarrow \mathbb {R}$ defined by

6.1 $$\begin{aligned} v(t,x)=\int _{x-\frac{\epsilon }{2}}^{x+\frac{\epsilon }{2}}U_\epsilon (t;u_0)(y)\textrm{d}y,\ \forall t\ge 0,\ x \in \mathbb {R} \end{aligned}$$

satisfies the following properties:

i) For each $t\ge 0$ and $x \in [0,1]$, we have the inequality $0\le v(t,x) \le \epsilon \Vert u_0 \Vert e^{bt}$;
ii) The function *v* is of class $C^\infty$ on $(0,\infty )\times [0,1]$, and for each $(t,x) \in (0,\infty )\times [0,1]$, it satisfies the partial differential equation
  6.2 $$\begin{aligned} \partial _t v(t,x)-D\partial _{xx}v(t,x)=\int _{x-\frac{\epsilon }{2}}^{x+\frac{\epsilon }{2}} F_\epsilon (U_\epsilon (t,u_0))(y)\textrm{d}y. \end{aligned}$$

### Proof

Let $\epsilon >0$ be given and fixed. Let $u_0\in X_+$ be given and set $u(t,x):=U_\epsilon (t,u_0)(x)$ for all $t\ge 0$ and $x \in [0,1]$. The property *i)* is a direct consequence of the definition of *v* in (6.1) and the estimate of the semiflow in (2.7). To prove *ii)* we recall that from Proposition 2.1 one has $u \in C^\infty ((0,+\infty )\times [0,1],\mathbb {R})$ and since $F_\epsilon$ is of class $C^\infty$ on *X* it comes that *v* is of class $C^\infty$ on $(0,+\infty )\times [0,1]$. Moreover, we have for each $(t,x)\in (0,+\infty )\times [0,1]$

$$\begin{aligned} \partial _t v(t,x)=\int _{x-\frac{\epsilon }{2}}^{x+\frac{\epsilon }{2}} \partial _t u(t,y)\textrm{d}y=\int _{x-\frac{\epsilon }{2}}^{x+\frac{\epsilon }{2}} \left[ D \partial _{yy}u(t,y)+F(u(t,\cdot ))(y) \right] \textrm{d}y \end{aligned}$$

and the result follows by using the equality

$$\begin{aligned} \int _{x-\frac{\epsilon }{2}}^{x+\frac{\epsilon }{2}} \partial _{yy}u(t,y) \textrm{d}y=\partial _{xx}v(t,x). \end{aligned}$$

$$\square $$

We give now the proof of Theorem 2.2.

### Proof of Theorem 2.2

The proof of Theorem 2.2 is divided into two steps. To this end, let $\epsilon >0$ be given and fixed. Let $u_0\in X_+$ be given and set $u(t,x):=U_\epsilon (t,u_0)(x)$ for all $t\ge 0$ and $x \in [0,1]$. Next, we consider the map $v: [0,+\infty )\times [0,1]\rightarrow \mathbb {R}$ defined in 6.1 that is

6.3 $$\begin{aligned} v(t,x):=\int _{x-\frac{\epsilon }{2}}^{x+\frac{\epsilon }{2}} u(t,y)\textrm{d}y,\ \forall t\ge 0,\ x \in [0,1] \end{aligned}$$

and we set

6.4 $$\begin{aligned} M_\epsilon :=\epsilon \max \left( \Vert u_0 \Vert ,\dfrac{b}{c}\right) \end{aligned}$$

where $c>0$ is the positive constant provided in Assumption 1.1.

**Step 1:** In this step we prove that

$$\begin{aligned} \Vert v(t, \cdot )\Vert \le M,\ \forall t\ge 0,\ \forall M>M_\epsilon . \end{aligned}$$

Before proceeding, we note that using Lemma 6.1, *v* if of class $C^\infty$ on $(0,+\infty )\times [0,1]$ so that $\partial _t v$ is bounded on any interval of the form $[a_0,a_1]\times [0,1]$ with $0<a_0< a_1$. Consequently, using the mean value theorem it follows that $t \mapsto \Vert v(t,\cdot )\Vert$ is continuous on $[0,+\infty )$. Next, we set

$$\begin{aligned} t_0:=\sup \Omega \ \text { with } \Omega :=\left\{ t>0: \Vert v(s, \cdot )\Vert < M,\ \forall s \in [0,t]\right\} . \end{aligned}$$

It is worth noting that $\Omega$ is nonempty because $\Vert v(0, \cdot )\Vert <M$ and $t \mapsto \Vert v(t,\cdot )\Vert$ is continuous on $[0,+\infty )$. In the following, we prove that $t_0=+\infty$. To do so, we argue by contradiction and assume that $t_0<+\infty$. Since $0<t_0<+\infty$ and $t \mapsto \Vert v(t,\cdot )\Vert$ is continuous on $[0,+\infty )$ we readily have

6.5 $$\begin{aligned} \Vert v(t_0,\cdot )\Vert =M \ \text { and } \ \Vert v(t,\cdot )\Vert \le M, \ \forall t\in [0,t_0). \end{aligned}$$

From where, there exists a sequence $(x_n)\subseteq [0,1]$ such that

6.6 $$\begin{aligned} v(t_0,x_n)\rightarrow M\, \text { as }\, n\rightarrow +\infty . \end{aligned}$$

Now consider the sequences defined for all $t\ge 0$ and $x\in [0,1]$ by

$$\begin{aligned} u_n(t,x)=u(t,x+x_n)\, \text {and}\, v_n(t,x)=v(t,x+x_n). \end{aligned}$$

As the sequences $(u_n)$ and $(v_n)$ are locally bounded in $C^k((0,\infty )\times [0,1])$ for all $k\in \mathbb {N}$, we infer by the standard parabolic estimates that $(u_n)$ and $(v_n)$ have subsequences which converge locally uniformly to $u_\infty \in C^\infty ((0,\infty )\times [0,1])$ and $v_\infty \in C^\infty ((0,\infty )\times [0,1])$. Thanks to the local uniform convergence, we can pass to the limit under the integral sign in (6.3) to obtain

$$\begin{aligned} v_\infty (t,x)=\int _{x-\frac{\epsilon }{2}}^{x+\frac{\epsilon }{2}}u_\infty (t,y)\textrm{d}y,\ \forall t>0,\ \forall x \in [0,1]. \end{aligned}$$

Since $(x_n)$ is bounded, it has a subsequence relabeled $(x_n)$, which converges to $x_\infty$. Thus, the uniform continuity of $x \mapsto \mu _m(x)$ on $\mathbb {R}$ implies that the sequence $x\mapsto \mu _{m,n}(x):=\mu _m(x+x_n)$ converges to $x\mapsto \mu _{m,\infty }(x)$ uniformly for *x* in compacts intervals of $\mathbb {R}$. Hence, we obtain for all $t\ge 0$ and $x\in [0,1]$

6.7 $$\begin{aligned} \partial _tv_\infty (t,x)=D\partial _{xx}v_\infty (t,x)+\int _{x-\frac{\epsilon }{2}}^{x+\frac{\epsilon }{2}}u_\infty (t,y) F_{\infty }(u_\infty (t,\cdot ))(y)\textrm{d}y, \end{aligned}$$

where $F_\infty : X \rightarrow X$ is given by

$$\begin{aligned} F_\infty (\varphi ):=b -\mu _{m,\infty } -\int _0^1K_\epsilon (\cdot -y)\varphi (y)\textrm{d}y,\ \forall \varphi \in X. \end{aligned}$$

In the following, we prove there exists $y_0 \in \left( -\frac{\epsilon }{2},\frac{\epsilon }{2}\right)$ such that $F_\infty (\varphi )(y_0)\ge 0$. To this end, we first observe that due to (6.5) and (6.6) we have

$$\begin{aligned} v_\infty (t_0,0)=M \ \text { and } 0\le v_\infty (t,x)\le M,\, \forall t\in [0,t_0),\ \forall x \in [0,1] \end{aligned}$$

providing that

6.8 $$\begin{aligned} \partial _tv_\infty (t_0,0)\ge 0\, \text { and }\, \partial _{xx}v_\infty (t_0,0)\le 0. \end{aligned}$$

Thus, we deduce from (6.7) and (6.8) that

6.9 $$\begin{aligned} \int _{-\frac{\epsilon }{2}}^{\frac{\epsilon }{2}}u_\infty (t_0,y) F_\infty (u_\infty (t_0,\cdot ))(y)\textrm{d}y=\partial _tv_\infty (t_0,0)-\partial _{xx}v_\infty (t_0,0)\ge 0. \end{aligned}$$

Next, we use (6.9) to prove that we cannot have $F_\infty (u_\infty (t_0,\cdot ))(y)<0$ for all $y\in \left( -\frac{\epsilon }{2},\frac{\epsilon }{2}\right)$. Indeed, if this is the case then using the fact that $u_\infty (t_0,y)\ge 0$ for all $y\in \mathbb {R}$ we obtain

6.10 $$\begin{aligned} \int _{-\frac{\epsilon }{2}}^{\frac{\epsilon }{2}}u_\infty (t_0,y) F_\infty (u_\infty (t_0,\cdot ))(y)\textrm{d}y\le 0 \end{aligned}$$

and therefore, (6.9)–(6.10) imply that

$$\begin{aligned} \int _{-\frac{\epsilon }{2}}^{\frac{\epsilon }{2}}u_\infty (t_0,y) F_\infty (u_\infty (t_0,\cdot ))(y)\textrm{d}y=0. \end{aligned}$$

Consequently, $u_\infty (t_0,y)=0$ for all $y \in \left( -\frac{\epsilon }{2},\frac{\epsilon }{2}\right)$ which gives the contradiction $v(t_0,0)>M$ and

$$\begin{aligned} v(t_0,0)=\int _{-\frac{\epsilon }{2}}^{\frac{\epsilon }{2}} u_\infty (t_0,y) \textrm{d}y=0. \end{aligned}$$

Therefore, we conclude that there exists $y_0\in \left( -\frac{\epsilon }{2},\frac{\epsilon }{2}\right)$ such that

6.11 $$\begin{aligned} 0\le F_\infty (u_\infty (t_0,\cdot ))(y_0)=b -\mu _{m,\infty }(y_0) -\int _0^1K_\epsilon (y_0-z)u(t_0,z)\textrm{d}z \end{aligned}$$

and we obtain the following contradiction

$$\begin{aligned} \begin{array}{llll} b\ge \mu _{m,\infty }(y_0) -\int _0^1K_\epsilon (y_0-z)u(t_0,z)\textrm{d}z& \ge & \int _{-\epsilon }^{\epsilon }\frac{1}{\epsilon } J\left( \frac{z}{\epsilon }\right) u_\infty (t_0,y_0-z)\textrm{d}z\\ & \ge & \frac{c}{\epsilon }\int _{-\frac{\epsilon }{2}}^{\frac{\epsilon }{2}}u_\infty (t_0,z)\textrm{d}z=\frac{cM}{\epsilon }>b. \end{array} \end{aligned}$$

**Step 2:** In this step, we complete the proof of the uniform boundedness of *u*. To do this, let $s\ge 1$, an arbitrary time. Using the maximum principle we obtain

6.12 $$\begin{aligned} 0\le u(s,x)\le w(s,x),\, \forall x\in [0,1], \end{aligned}$$

where *w* denotes the solution of $\partial _tw=D\partial _{xx}w+bw$ with the initial condition $w(s-1,\cdot )=u(s-1;\cdot )$ at time $s-1$. Note that the map *w* satisfies the following expression

6.13 $$\begin{aligned} w(t,x)=e^{b(t-t_0)}T_A(t-t_0)w(t_0,x),\ \ \forall t\ge t_0\ge 0,\ x\in [0,1]. \end{aligned}$$

Using the formula of the semigroup $\{T_{A}(t)\}_{t\ge 0}$ in (2.5) and substituting *t* and $t_0$ with *s* and $s-1$ respectively in (6.13), we obtain the following equality for all $x \in [0,1]$

6.14 $$\begin{aligned} w(s,x)=e^{b}\sum _{k\in \mathbb {Z}}\frac{1}{\sqrt{4\pi D}}\int _0^1e^{-\frac{(x-y-k)^2}{4D}}u(s-1,y)\textrm{d}y. \end{aligned}$$

Since $y \mapsto u(s-1,y)$ is 1-periodic, we deduce from (6.14) that

6.15 $$\begin{aligned} w(s,x)=\dfrac{e^b}{\sqrt{4\pi D}}\sum _{k\in \mathbb {Z}}\int _{(k-\frac{1}{2})\epsilon }^{(k+\frac{1}{2})\epsilon }e^{-\frac{y^2}{4D}}u(s-1,x-y)\textrm{d}y,\ \forall x \in [0,1]. \end{aligned}$$

Thus, from (6.15), we have for each $x\in [0,1]$

6.16 $$\begin{aligned} \begin{array}{llll} w(s,x)& \le & \dfrac{e^b}{\sqrt{4\pi D}}\sum _{k=-\infty }^{0}\int _{(k-\frac{1}{2})\epsilon }^{(k+\frac{1}{2})\epsilon }e^{-\frac{k^2\epsilon ^2}{4D}}u(s-1,x-y)\textrm{d}y\\ & & +\dfrac{e^b}{\sqrt{4\pi D}}\sum _{k=1}^{+\infty }\int _{(k-\frac{1}{2})\epsilon }^{(k+\frac{1}{2})\epsilon }e^{-\frac{(k-1)^2\epsilon ^2}{4D}}u(s-1,x-y)\textrm{d}y. \end{array} \end{aligned}$$

Using (6.3) and (6.16), we obtain

6.17 $$\begin{aligned} \begin{array}{llll} w(s,x)& \le & \dfrac{e^b}{\sqrt{4\pi D}}\sum _{k=-\infty }^{0}e^{-\frac{k^2\epsilon ^2}{4D}}v(s-1,x-k\epsilon )\\ & & +\dfrac{e^b}{\sqrt{4\pi D}}\sum _{k=1}^{+\infty }e^{-\frac{(k-1)^2\epsilon ^2}{4D}}v(s-1,x-k\epsilon ). \end{array} \end{aligned}$$

According to step 1 of this proof, we have $v(t,x)\le M_\epsilon$ for all $t\ge 0$, $x\in \mathbb {R}$. Therefore, we deduce from (6.17) that for each $x \in [0,1]$

6.18 $$\begin{aligned} w(s,x) &  \le \dfrac{e^b}{\sqrt{4\pi D}}M_\epsilon \left( \sum _{k=-\infty }^{0}e^{-\frac{k^2\epsilon ^2}{4D}}+\dfrac{e^b}{\sqrt{4\pi D}}\sum _{k=1}^{+\infty }e^{-\frac{(k-1)^2\epsilon ^2}{4D}}\right) \nonumber \\  &  =\dfrac{2e^b}{\sqrt{4\pi D}}M_\epsilon \sum _{k=0}^{\infty }e^{-\frac{k^2\epsilon ^2}{4D}}. \end{aligned}$$

Next, we obtain from (6.18) that

$$\begin{aligned} &  w(s,x)\le 2\dfrac{e^bM_\epsilon }{\sqrt{4\pi D}}\left( 1+\sum _{k=1}^{\infty }\int _{k-1}^{k}e^{-\frac{y^2\epsilon ^2}{4D}}\textrm{d}y\right) =2\dfrac{e^bM_\epsilon }{\sqrt{4\pi D}}\left( 1+\int _0^\infty e^{-\frac{y^2\epsilon ^2}{4D}}\textrm{d}y\right) , \\  &  \qquad \ \forall x \in [0,1], \end{aligned}$$

hence

6.19 $$\begin{aligned} w(s,x)\le \dfrac{e^bM_\epsilon }{\sqrt{\pi D}}\left( 1+\frac{\sqrt{\pi D}}{\epsilon }\right) ,\ \forall x \in [0,1]. \end{aligned}$$

Combining (6.12) and (6.19), we obtain the following inequality for all $s\ge 1$ and $x\in [0,1]$:

6.20 $$\begin{aligned} u(s,x)\le \dfrac{e^bM_\epsilon }{\sqrt{\pi D}}\left( 1+\frac{\sqrt{\pi D}}{\epsilon }\right) . \end{aligned}$$

Finally, combining (2.7), (6.4), and (6.20) we obtain

$$\begin{aligned} 0\le u(t,x)\le e^b\max \left( \Vert \varphi \Vert ,\frac{b}{c}\right) \left( 1+\frac{\epsilon }{\sqrt{\pi D}}\right) \, \forall t\ge 0,\ \forall x\in [0,1]\ \text { and } \forall \epsilon \ge 0 \end{aligned}$$

and the proof is completed. $\square$

The theorem above demonstrates that for any positive initial condition in $X_+$, the solution of (2.1) is uniformly bounded. In the following, we will demonstrate the existence of equilibrium states for equation (2.1) and analyze the stability of these equilibrium states.

## Proof of Theorems 2.3 and 2.4: dynamics of mosquitoes

In this section, we aim to investigate the existence of a globally asymptotically stable equilibrium state for the parameterized system (2.1). To begin, we establish the existence of $(\mu _1,\phi _1)$ satisfying the following eigenvalue problem:

7.1 $$\begin{aligned} {\left\{ \begin{array}{ll} L\phi _1+b \phi _1=\mu _1 \phi _1\, \text {in}\ \mathbb {R}\\ \phi _1\in X,\, \phi _1>0,\, \Vert \phi _1\Vert =1 \end{array}\right. } \end{aligned}$$

where $\mu _1$ represents the principal eigenvalue of $L+b$, and establish its connection to the mosquito’s reproductive rate $\mathcal {T}_0$ given in (2.12). It’s worth noting that, by using the results in Thieme (2009), we obtain the following sign equality:

$$\begin{aligned} \text {sgn}(b+\textsf{s}(L))=\text {sgn}(\mathcal {T}_0-1) \end{aligned}$$

with $\textsf{s}(L)$ the spectral bound of *L* defined in (2.11). Moreover, we note that *L* is a generator of an analytic semigroup as a bounded perturbation of the Laplace operator *A*. Therefore, we have $\textsf{s}(L)=\omega (L)$, with $\omega (L)$ the growth bound of the semigroup generated by *L*.

### Proposition 7.1

Let Assumption 1.1 be satisfied. Then, $T_{L}(t)$ is compact for each $t>0$ and $b+\textsf{s}(L)$ is the principal eigenvalue of $L+b$ with a positive eigenvector $\phi _1\in \text {int}(X_+)$ normalized to 1. Specifically, if we set $\mu _1=b+\textsf{s}(L)$, then $(\mu _1,\phi _1)$ represents the unique solution to (7.1).

### Proof

Firstly, we observe that $b+\textsf{s}(L)=\textsf{s}(b+L)$. Next, recalling that $L=A-\mu _m$ we have for $\lambda >0$ large enough such that $\lambda +\mu _m(x)>0$ for all $x \in [0,1]$

7.2 $$\begin{aligned} T_{L}(t)=e^{-\lambda t} T_{A}(t) +\int _0^t e^{-\lambda (t-s)} T_{A}(t-s)(\lambda +\mu _m) \textrm{d}s,\ \forall t\ge 0. \end{aligned}$$

Since $\{ T_{A}(t)\}_{t\ge 0}$ is positive irreducible it follows from (7.2) that $\{ T_{L}(t)\}_{t\ge 0}$ is also positive irreducible and therefore $(\textsf{s}(L),+\infty )$ belongs in the resolvent set of *L*. Moreover, *L* is a generator of a compact semigroup as a bounded perturbation of a generator of compact semigroup (Smith and Thieme 2011). Consequently, *L* has a compact resolvent. Since $\lambda \in (\textsf{s}(L),+\infty ) \mapsto b (\lambda -L)^{-1}$ is continuous in the operator norm topology and $b (\lambda -L)^{-1}$ is compact it follows that $\lambda \in (\textsf{s}(L),+\infty ) \mapsto \textsf{r} (b (\lambda -L)^{-1})=b\textsf{r} ((\lambda -L)^{-1})$ is also continuous (Degla 2008). Finally, using the fact that $\textsf{s}(L)$ belongs in the spectrum $\sigma (L)$ of *L* and

$$\begin{aligned} \lim _{\lambda \rightarrow +\infty } (\lambda -L)^{-1}=0 \ \text { and } \textsf{r}((\lambda -L)^{-1})=\dfrac{1}{\inf _{\mu \in \sigma (L)}\vert \lambda -\mu \vert },\, \forall \lambda \in \rho (L), \end{aligned}$$

it comes that

$$\begin{aligned} \lim _{\lambda \rightarrow +\infty } b\textsf{r} ((\lambda -L)^{-1})=0 \ \text { and } \lim _{\lambda \rightarrow \textsf{s}(L)^-}=b\textsf{r} ((\lambda -L)^{-1})=+\infty . \end{aligned}$$

Hence, we deduce that there exists $\lambda _1>\textsf{s}(L)$ and $\lambda _2>\textsf{s}(L)$ such that $b\textsf{r} ((\lambda _1-L)^{-1})>1>b\textsf{r} ((\lambda _2-L)^{-1})$. Since $\{ T_{L}(t)\}_{t\ge 0}$ is positive irreducible it follows that $(\lambda -L)^{-1}$, $\lambda >\textsf{s}(L)$ is also positive irreducible and the proof is completed by using Thieme (1997, Theorem 4.7). $\square$

### Equilibrium without mosquitoes

We study the existence of equilibrium states and the long-term behavior of the solution of equation (2.1) when $\mu _1\le 0$ that is $\mathcal {T}_0\le 1$. The theorem below gives a sufficient condition to ensure the convergence of the solution of problem (2.1).

#### Theorem 7.2

Let Assumption 1.1 be satisfied and $\mu _1\le 0$. Then (2.1) has no positive equilibrium. Furthermore, if $\mu _1 \le 0$, then all non-negative solutions converge to zero as $t \rightarrow \infty$.

#### Proof

Assume that $\mu _1\le 0$. We first prove that 0 is the unique nonnegative equilibrium of (2.1). Let $\phi _1\in X$ be the unique positive solution of (7.1) associated with the principal eigenvalue $\mu _1$ and let *u* be a positive, bounded stationary solution of equation (2.1). For each $\epsilon \in [0,\epsilon _0]$ and $\gamma >0$, $\gamma \phi _1$ is a supersolutions of the problem

7.3 $$\begin{aligned} \left\{ \begin{array}{llll} -Du''(x)-\left( b-\mu _m-\int _0^{1}K_\epsilon (x-y)u(y)\textrm{d}y\right) u(x)=0,\; x\in (0,1)\\ u(0)=u(1)\\ u'(0)=u'(1). \end{array}\right. \end{aligned}$$

Since $\phi _1$ is strictly positive, bounded and *u* is bounded, one can define

$$\begin{aligned} \gamma ^*=\inf \{\gamma>0: \gamma \phi _1>u\ \text {in}\ \mathbb {R}\}\ge 0. \end{aligned}$$

Assume that $\gamma ^*>0$ and set $\varphi =\gamma ^*\phi _1-u$ which nonnegative by definition of $\gamma ^*$. Since the map $\varphi$ is 1-periodic and continuous, there $\overline{x}\in [0,1]$ such that $\varphi (\overline{x})=0$. Moreover, we have

$$\begin{aligned} {\left\{ \begin{array}{ll} -D\varphi ''-(b-\mu _m)\varphi =\gamma ^*\phi _1+(K_\epsilon *u)u\ge 0\\ \varphi \ge 0,\ \text {in}\,\mathbb {R}, \end{array}\right. } \end{aligned}$$

so that the strong maximum principle ensures that $\varphi \equiv 0$ *i.e* $\gamma ^*\phi _1=u$. Hence, we can replace *u* by $\gamma ^*\phi _1$ in (7.3) to obtain

$$\begin{aligned} 0=-D(\gamma ^*\phi _1)''-\gamma ^*\left( b-\mu _m-\gamma ^*\int _0^{1}K_\epsilon (x-y)\phi _1(y)\textrm{d}y\right) \phi . \end{aligned}$$

Therefore, using (7.1), we obtain the following contradiction

$$\begin{aligned} 0= &  -D(\gamma ^*\phi _1)''-\gamma ^*\left( b-\mu _m-\gamma ^*\int _0^{1}K_\epsilon (x-y)\phi _1(y)\textrm{d}y\right) \phi _1\\ = &  -\mu _1\gamma ^*\phi _1+(\gamma ^*)^2\int _0^{1}K_\epsilon (x-y)\phi _1(y)\textrm{d}y>0. \end{aligned}$$

Consequently, we have $\gamma ^*=0$ that is $u=\gamma ^* \phi _1 \equiv 0$.

We will now show that when $\mu _1\le 0$, then the equilibrium state $\bar{u} \equiv 0$ of (2.1) is globally asymptotically stable in $X_+$. The proof will be done in two cases.

**If** $\mu _1<0$: Let $u_0\in X_+$ be given and fixed. The function $\psi$ defined by $\psi _n(t,x)=n e^{\mu _1t}\phi _1(x)$, with $n\in \mathbb {N}$, satisfies the following inequality

7.4 $$\begin{aligned} \partial _t\psi _n(t,x)-D\partial _{xx}\psi _n(t,x)-\left( b-\mu _m(x)-\int _0^{1}K_\epsilon (x-y)\psi _n(t,y)\textrm{d}y\right) \psi _n(t,x)>0 \end{aligned}$$

for every $(t,x)\in (0,\infty )\times (0,1)$ and $\epsilon \in [0,\epsilon _0]$. Since $\phi$ is positive and $u_0$ is bounded, there exists an integer $n\in \mathbb {N}$ such that $\psi _n(0,x)\ge U_\epsilon (0;u_0)(x)$ for all $x\in [0,1]$ and $\epsilon \in [0,\epsilon _0]$. Thus, from (7.4), $\psi$ becomes a super-solution of (2.4). Therefore, we obtain

$$\begin{aligned} U_\epsilon (t;u_0)(x)\le \psi _n(t,x)\ \text { for every } (t,x,\epsilon )\in \mathbb {R}_+\times \mathbb {R}\times [0,\epsilon _0]. \end{aligned}$$

Moreover, since $\mu _1<0$, we have $\psi _n(t,x)\rightarrow 0$ exponentially as $t\rightarrow \infty$, implying that all solutions of (2.1) decay exponentially toward zero as well.

**If** $\mu _1=0$: Let $u_0\in X_+$ be given and fixed. Let consider the functional *V* defined by

$$\begin{aligned} V[u]=\int _0^1 u(x)\phi _1(x)\textrm{d}x,\, \forall u\in X. \end{aligned}$$

By multiplying $\phi _1$ in (2.1) and then integrating by parts, we obtain

7.5 $$\begin{aligned} \frac{\textrm{d}V[U(t;u_0)]}{\textrm{d}t} &  =\int _0^1 \partial _t U_\epsilon (t;u_0)(x)\phi _1(x)\textrm{d}x \nonumber \\ &  =\int _0^1U_\epsilon (t;u_0)(x)[D\phi _1''(x)+(b-\mu _m(x))\phi _1(x)]\textrm{d}x \nonumber \\ &  \quad -\int _0^1U_\epsilon (t;u_0)(x)\int _0^1 K_\epsilon (x-y)U_\epsilon (t,u_0)(y)\textrm{d}y))\phi _1(x))\textrm{d}x.\nonumber \\ \end{aligned}$$

Since $\mu _1=0$, it follows from (7.1) and (7.5) that

$$\begin{aligned} \frac{\textrm{d}V[U(t;u_0)]}{\textrm{d}t}= -\int _0^1U_\epsilon (t;u_0)(x)\left( \int _0^1 K_\epsilon (x-y)U_\epsilon (t,u_0)(y)\textrm{d}y\right) \phi _1(x))\textrm{d}x<0. \end{aligned}$$

Consequently, $U_\epsilon (t;u_0)$ declines toward to 0 as $t\rightarrow \infty$. $\square$

### Equilibrium with mosquitoes

We investigate the existence of stationary solutions of (2.1) when $\mu _1 > 0$. Initially, we analyze problem (2.1) with $\epsilon = 0$. Subsequently, we extend the perturbation results established in Smith and Waltman (1999) (also referenced in Magal 2009) to prove Theorem 2.4. The following lemma can be proved by using similar arguments in Lam and Lou (2022)

#### Lemma 7.3

Let Assumption 1.1 be satisfied. Consider the problem

7.6 $$\begin{aligned} {\left\{ \begin{array}{ll} \partial _t u(t,x)=D\partial _{xx}u(t,x)+(b-\mu _m(x)-u(t,x))u(t,x), \ t>0,\ x \in (0,1)\\ u(t,0)=u(t,1), \ t>0\\ \partial _x u(t,0)=\partial _x u(t,1), \ t>0\\ u(0,\cdot )=u_0\in X_+. \end{array}\right. } \end{aligned}$$

If $\mu _1>0$, then (7.6) has a unique positive equilibrium $\varphi _0\in \text {int}(X_+)$. Moreover, the equilibrium $\varphi _0$ is globally asymptotically stable among all nonnegative, nontrivial solutions.

In the following, we define $\{U_0(t)\}_{t\ge 0}$ as the continuous semiflow generates by (7.6) on $X_+$. We will deduce the behavior of (2.1) for $\epsilon \ll 1$.

#### Lemma 7.4

Let Assumption 1.1 be satisfied. Then the map $(\epsilon ,u)\mapsto K_\epsilon *u$ is continuous from $[0,\epsilon _0]\times X$ into *X*.

#### Proof

Consider $\epsilon ,\eta \in [0,\epsilon _0]$, $u,v\in X_+$ and observe that

7.7 $$\begin{aligned} \Vert K_{\epsilon }*u-K_\eta *v\Vert \le \Vert (K_{\epsilon }-K_\eta )*u\Vert +\Vert u-v\Vert \end{aligned}$$

where we have used the equality $\int _0^1 K_{\epsilon }(y)\textrm{d}y=1$ for $\epsilon \ge 0$. From (7.7) it follows that the continuity of $(\epsilon ,u)\mapsto K_\epsilon *u$ can be obtained from the continuity of $\epsilon \in [0,\epsilon _0] \mapsto K_{\epsilon }*u$ for any fixed $u \in X_+$. In the remainder of the proof, with $u \in X_+$ fixed, we distinguish the case $\eta =0$ and $\eta \in (0,\epsilon _0]$.

**Continuity at** $\eta =0$: Let $u \in X_+$ be given and recall that $K_\eta * u=u$ for $\eta =0$. Then we have for each $\epsilon \in (0,\epsilon _0]$ and $x \in [0,1]$

$$\begin{aligned} \begin{array}{lll} (K_\epsilon *u-u)(x)& =& \int _0^{1}[u(x-y)-u(x)]K_\epsilon (y)\textrm{d}y\\ & =& \int _{-\infty }^{+\infty }\frac{1}{\epsilon }J\left( \dfrac{y}{\epsilon }\right) [u(x-y)-u(x)]\textrm{d}y, \end{array} \end{aligned}$$

so that for each $r>0$

$$\begin{aligned} \begin{array}{lll} \vert (u-K_\epsilon *u)(x)\vert & \le & \displaystyle \sup _{\vert y \vert \le r} \vert u(x-y)-u(y)\vert + 2\sup _{y \in \mathbb {R}} \vert u(y) \vert \int _{\vert y \vert \ge r} \frac{1}{\epsilon }J\left( \dfrac{y}{\epsilon }\right) \textrm{d}y \\ & \le & \displaystyle \sup _{\vert y \vert \le r} \vert u(x-y)-u(y)\vert + 2\sup _{y \in \mathbb {R}} \vert u(y) \vert \int _{\vert y \vert \ge \frac{r}{\epsilon }} J\left( y\right) \textrm{d}y. \end{array} \end{aligned}$$

Next, using the uniform continuity of $u \in [0,1]$ we deduce that for each $n \in \mathbb {N}$ there exists $r_n>0$ small enough such that

$$\begin{aligned} \vert (u-K_\epsilon *u)(x)\vert \le \dfrac{1}{n+1}+2\sup _{y \in \mathbb {R}} \vert u(y) \vert \int _{\vert y \vert \ge \frac{r_n}{\epsilon }} J\left( y\right) \textrm{d}y,\ \forall x \in [0,1] \end{aligned}$$

providing that

$$\begin{aligned} \limsup _{\epsilon \rightarrow 0^+} \sup _{x \in [0,1]} \vert (u-K_\epsilon *u)(x)\vert \le \dfrac{1}{n+1},\ \forall n \ge 0 \end{aligned}$$

and the result follows.

**Continuity for** $\eta \in (0,\epsilon _0]$: Let $u \in X_+$ and $\eta \in (0,\epsilon _0]$ be given and fixed. Note that for each $\epsilon \in (0,\epsilon _0)$ and $x\in [0,1]$ we have

7.8 $$\begin{aligned} \vert (K_\epsilon *u- K_{\eta }*u)(x)\vert\le &  \int _\mathbb {R}\left| \frac{1}{\eta }J\left( \frac{y}{\eta }\right) - \frac{1}{\epsilon }J\left( \frac{y}{\epsilon }\right) \right| \vert u(x-y)\vert \textrm{d}y \nonumber \\\le &  2\sup _{y \in \mathbb {R}} \vert u(y) \vert \int _\mathbb {R}\left| \frac{\epsilon }{\eta }J\left( \frac{\epsilon }{\eta }y\right) -J(y)\right| \textrm{d}y. \end{aligned}$$

Therefore, setting $\xi =\frac{\epsilon }{\eta }-1$ it follows from (7.8) that

7.9 $$\begin{aligned} \Vert K_\epsilon *u- K_{\eta }*u\Vert\le &  \Vert u\Vert \int _\mathbb {R}\left| (\xi +1)J((\xi +1)y)-J(y)\right| \textrm{d}y \nonumber \\\le &  \dfrac{\vert \xi \vert }{\xi +1}\Vert u\Vert \int _\mathbb {R}J(y)\textrm{d}y+\Vert u\Vert \int _\mathbb {R}\left| J(\xi y+y)-J(y)\right| \textrm{d}y.\nonumber \\ \end{aligned}$$

Moreover for each $r>0$ we obtain from (7.9)

7.10 $$\begin{aligned} \Vert K_\epsilon *u- K_{\eta }*u\Vert\le &  \Vert u\Vert \bigg (\dfrac{\vert \xi \vert }{\xi +1}\int _\mathbb {R}J(y)\textrm{d}y\nonumber \\ &  +\int _{\vert y\vert \le r}\left| J(\xi y+y)-J(y)\right| \textrm{d}y+\int _{\vert y\vert \ge r}\left| J(\xi y+y)-J(y)\right| \textrm{d}y\bigg )\nonumber \\\le &  \Vert u\Vert \bigg (\dfrac{\vert \xi \vert }{\xi +1}+\int _{\vert y\vert \le r}\left| J(\xi y+y)-J(y)\right| \textrm{d}y\nonumber \\ &  +\frac{1}{\xi +1}\int _{\vert y\vert \ge (1+\xi )r} J(y)\textrm{d}y+\int _{\vert y\vert \ge r}J(y) \textrm{d}y\bigg ). \end{aligned}$$

Since $J\in L^1(\mathbb {R})$ and bounded, the restriction on a ball $B(0,r)\subset \mathbb {R}$ of the map $\xi \mapsto J((\xi +1)\cdot )$ defined from $\mathbb {R}$ into $L^1(\mathbb {R})$ is continuous. Thus, we deduce from (7.10) that

7.11 $$\begin{aligned} \limsup _{\epsilon \rightarrow \eta }\Vert K_\epsilon *u- K_{\eta }*u\Vert \le 2\int _{\vert y\vert \ge r}J(y) \textrm{d}y \end{aligned}$$

and the result follows by letting *r* goes to $+\infty$ in (7.11). $\square$

#### Proposition 7.5

Let Assumption 1.1 be satisfied. Then for every bounded subset $B\subset X_+$, $\tau >0$ and $\tau _0 \in (0,\tau ]$ the set

$$\begin{aligned} \mathcal {B}:=\{U_\epsilon (t;u_0): t\in [\tau _0,\tau ],\, u_0\in B,\, \epsilon \in [0,\epsilon _0]\} \end{aligned}$$

is relatively compact.

#### Proof

Let $\tau >0$, $\tau _0 \in (0,\tau ]$ and *B* be a bounded subset of $X_+$. To prove that $\mathcal {B}$ is relatively compact, we prove that for each $\alpha \in (0,\tau _0)$ there exists a relatively compact set $\mathcal {B}_\alpha$ and a constant $\kappa >0$ such that $\mathcal {B}\subset \mathcal {B}_\alpha +\kappa \alpha \overline{B_X(0,1)}$ with $\overline{B_X(0,1)}$ the unit closed ball in *X*.

Recall that for all $t\ge 0$, $u_0\in X_+$ and $\epsilon \in [0,\epsilon _0]$, we have

$$\begin{aligned} \Vert U_\epsilon (t;u_0)\Vert \le e^{bt}\Vert u_0\Vert . \end{aligned}$$

This implies that the set $\{U_\epsilon (t;u_0): t\in [\tau _0,\tau ],\ u_0\in B,\ \epsilon \in [0,\epsilon _0]\}$ is bounded. Specifically, there exists $\kappa _0:=\kappa _0(\tau , B)>0$ such that

7.12 $$\begin{aligned} U_\epsilon (t;u_0)\in B_X(0,\kappa _0),\ \forall t\in [\tau _0,\tau ],\ u_0\in B\ \text { and } \epsilon \in [0,\epsilon _0]. \end{aligned}$$

Since $F_\epsilon$ is Lipschitz for any bounded subset of $X_+$ uniformly in $\epsilon$ and $F_\epsilon (0)=0$, we deduce from (7.12) that there exists $\kappa >0$ such that

7.13 $$\begin{aligned} F_\epsilon (U_\epsilon (t;u_0))\in B_X(0,\kappa ), \forall t\ge 0,\ \epsilon \in [0,\epsilon _0] \text { and } u_0\in B. \end{aligned}$$

Next, using the semiflow properties, we have for every $t\in [\tau _0,\tau ]$, $\alpha \in (0,\tau _0)$ and $u_0\in B$

7.14 $$\begin{aligned} U_\epsilon (t;u_0)=T_A(\alpha )U_\epsilon (t-\alpha ;u_0)+\int _{t-\alpha }^tT_A(t-s)F_\epsilon (U_\epsilon (s;u_0))\textrm{d}s \end{aligned}$$

and combining (2.6) and (7.13) we obtain

7.15 $$\begin{aligned} \left\| \int _{t-\alpha }^tT_A(t-s)F_\epsilon (U_\epsilon (s;u_0))\textrm{d}s\right\| \le \kappa \alpha . \end{aligned}$$

Setting

$$\begin{aligned} \mathcal {B}_\alpha =\overline{\{T_A(\alpha )U_\epsilon (t-\alpha ;u_0): t\in [\tau _0,\tau ],\, u_0\in B,\, \epsilon \in [0,\epsilon _0]\}}, \end{aligned}$$

it follows from (7.14) and (7.15), that

$$\begin{aligned} \mathcal {B}\subset \mathcal {B}_\alpha +\kappa \alpha \overline{B_X(0,1)}. \end{aligned}$$

Since the set $\{U_\epsilon (t-\alpha ;u_0): t\in [\tau _0,\tau ],\ u_0\in B,\ \epsilon \in [0,\epsilon _0]\}$ is bounded and the operator $T(\alpha )$ is compact for each $\alpha \in (0,\tau _0)$, we deduce that $\mathcal {B}_\alpha$ is compact for each $\alpha \in (0,\tau _0)$. The proof is completed. $\square$

#### Proposition 7.6

Let Assumption 1.1 be satisfied. Then the following properties hold:

i) The nonlinear map $F_\epsilon : X\rightarrow X$ is differentiable on *X* and $(\epsilon ,u)\mapsto D_uF_\epsilon (u)$ is continuous from $(0,\epsilon _0]\times X_+$ into $\mathcal {L}(X)$.
ii) For each bounded set $B\subset X_+$ and $\tau >0$, $U_\epsilon (t;\varphi ) \rightarrow U_0(t;\varphi )$ as $\epsilon \rightarrow 0$ uniformly in $(t,\varphi )\in [0,\tau ]\times B$.

#### Proof

Using Lemma 7.4 it is easly proved that for each $\epsilon \in [0,\epsilon _0]$ the map $u\mapsto F_\epsilon (u)$ is differentiable with respect to $u \in X_+$ with

7.16 $$\begin{aligned} D_uF_\epsilon (u).h=(b-\mu _m-K_\epsilon *u)h-(K_\epsilon *h)u,\ \forall h\in X\, \text {and}\, \epsilon \in [0,\epsilon _0]. \end{aligned}$$

Let $\epsilon ,\eta \in [0,\epsilon _0]$ be given. Observe that the continuity of $(\epsilon ,u)\mapsto D_uF_\epsilon (u)$ directly follows from Lemma 7.4. Indeed from (7.16), for all $u,v\in X_+$ and $h\in X$, we have:

7.17 $$\begin{aligned} \Vert D_uF_{\epsilon }(u+v)\cdot h-D_uF_{\eta }(u)\cdot h\Vert= &  \Vert [K_{\epsilon }*u-K_{\eta }*u]h+[K_{\epsilon }*h-K_{\eta }*h]u \nonumber \\ \nonumber \\ &  -[K_{\epsilon }*v]h-[K_{\epsilon }*h]v\Vert \nonumber \\ \nonumber \\\le &  \Vert K_{\epsilon }*u-K_{\eta }*u\Vert \Vert h\Vert +\Vert v\Vert \Vert h\Vert . \end{aligned}$$

Using (7.17), we obtain

$$\begin{aligned} \Vert D_uF_{\epsilon }(u+v)-D_uF_{\eta }(u)\Vert _{\mathcal {L}(X)}\le \Vert K_{\epsilon }*u-K_{\eta }*u\Vert +\Vert v\Vert \end{aligned}$$

and thus, the result follows.

We proceed to prove assertion ii). To this end, let *B* be a bounded subset of $X_+$ and $\tau >0$ a given constant. Recall the semiflow $U_\epsilon$ generated by (2.1) for all $u_0\in X_+$ and $\epsilon \in [0,\epsilon _0]$, defined by

7.18 $$\begin{aligned} U_\epsilon (t;u_0)=T_A(t)u_0+\int _0^tT_A(t-s)F_\epsilon (U_\epsilon (s;u_0))\textrm{d}s,\ \forall t>0. \end{aligned}$$

Let $u_0\in B$ and $t\in [0,\tau ]$ be given. From (7.18), we have

$$\begin{aligned} \begin{array}{rllll} U_\epsilon (t;u_0)-U_0(t;u_0)& =\int _0^tT_A(t-s)[F_\epsilon (U_\epsilon (s;u_0))-F_0(U_0(s;u_0))]\textrm{d}s \\ \\ & =\int _0^tT_A(t-s)[F_\epsilon (U_\epsilon (s;u_0))-F_\epsilon (U_0(s;u_0))]\textrm{d}s\\ \\ & \quad +\int _0^tT_A(t-s)[F_\epsilon (U_0(s;u_0))-F_0(U_0(s;u_0))]\textrm{d}s. \end{array} \end{aligned}$$

Next, note that using (1.3) and (2.3) we get

$$\begin{aligned} F_\epsilon (U_0(s;u_0))-F_0(U_0(s;u_0))=(K_\epsilon * U_0(s;u_0)-U_0(s;u_0)) U_0(s;u_0),\ \forall s \in [0,t]. \end{aligned}$$

Thus, using the inequality $\Vert T_A(t)\Vert \le 1$ for all $t\in [0,\tau ]$, we obtain for each $\beta \in (0,\tau )$

$$\begin{aligned} \begin{array}{rlllll} \Vert U_\epsilon (t;u_0)-U_0(t;u_0)\Vert \le & \displaystyle \int _0^t\Vert F_\epsilon (U_\epsilon (s;u_0))-F_\epsilon (U_0(s;u_0))\Vert \textrm{d}s \\ \\ & + \displaystyle \int _0^\beta \Vert F_\epsilon (U_0(s;u_0))-F_0(U_0(s;u_0))\Vert \textrm{d}s \\ \\ & + \displaystyle \int _\beta ^t \Vert K_\epsilon *U_0(s;u_0)-U_0(s;u_0)\Vert \Vert U_0(s;u_0)\Vert \textrm{d}s. \end{array} \end{aligned}$$

Next, since the function $F_\epsilon$ is Lipschitz, we infer from Theorem 2.2 that there exists $\gamma >0$ such that for every $t\in [0,\tau ]$, $u_0\in B$ and $\epsilon \in [0,\epsilon _0]$, we have:

7.19 $$\begin{aligned} \int _0^t\Vert F_\epsilon (U_\epsilon (s;u_0))-F_\epsilon (U_0(s;u_0))\Vert \textrm{d}s\le \gamma \int _0^t\Vert U_\epsilon (s;u_0)-U_0(s;u_0)\Vert \textrm{d}s.\nonumber \\ \end{aligned}$$

From Theorem 2.2, there exists $\kappa '>0$ such that for all $u_0\in B$ and $\epsilon \in [0,\epsilon _0]$,

7.20 $$\begin{aligned} \int _0^\beta \Vert F_\epsilon (U_0(s;u_0))- F_0(U_0(s;u_0))\Vert \textrm{d}s \le \beta \kappa '. \end{aligned}$$

We can also deduce from Proposition 7.5 and Lemma 7.4 that for all $\widehat{\beta }>0$, there exists $\hat{\epsilon }\in (0,\epsilon _0)$ such that for every $u_0\in B$, $s\in [\beta ,\tau ]$ and $\epsilon \in (0,\hat{\epsilon })$ we have

$$\begin{aligned} \Vert K_\epsilon *U_0(s;u_0)-U_0(s;u_0)\Vert \Vert U_0(s;u_0)\Vert \le \widehat{\beta }. \end{aligned}$$

Consequently, we obtain for every $u_0\in B,\, t\in [0,\tau ]\,\text {and}\; \epsilon \in (0,\hat{\epsilon })$

7.21 $$\begin{aligned} \int _\beta ^t\Vert U_0(s;u_0)\Vert \Vert K_\epsilon *U_0(s;u_0)-U_0(s,u_0)\Vert \textrm{d}s\le \widehat{\beta }(\tau -\beta ). \end{aligned}$$

Combining (7.19), (7.20) and (7.21), we obtain for all $u_0\in B,\,t\in [0,\tau ]\,\text {and}\;\epsilon \in (0,\hat{\epsilon })$:

$$\begin{aligned} \Vert U_\epsilon (t;u_0)-U_0(t;u_0)\Vert \le \gamma \int _0^t\Vert U_\epsilon (s;u_0)-U_0(s;u_0)\Vert \textrm{d}s+\beta \kappa +\widehat{\beta }(\tau -\beta ). \end{aligned}$$

Finally setting $\bar{\beta }=\beta \kappa +\widehat{\beta }(\tau -\beta )$ and applying Gronwall’s lemma, we obtain

$$\begin{aligned} \Vert U_\epsilon (t;u_0)-U_0(t;u_0)\Vert \le \bar{\beta }e^{\tau \kappa },\,u_0\in B,\,t\in [0,\tau ]\,\text {and}\;\epsilon \in (0,\hat{\epsilon }). \end{aligned}$$

Since $\bar{\beta }$ is arbitrary, we conclude

$$\begin{aligned} \lim _{\epsilon \rightarrow 0}\sup _{(t,u_0)\in [0,\tau ]\times B}\Vert U_\epsilon (t;u_0)-U_0(t;u_0)\Vert =0, \end{aligned}$$

which completes the proof of ii) and thus the proof of the proposition. $\square$

Using Proposition 7.6, Thieme (1990, Theorem 3.4), and Henry et al. (2006, Theorem 3.4.4) we obtain the following result.

#### Lemma 7.7

Let Assumption 1.1 be satisfied and $\mu _1>0$. Then the following properties hold:

i) For each $t\ge 0$ and $\epsilon \in [0,\epsilon _0]$, the map $u\mapsto U_\epsilon (t;u)$ is differentiable on $X_+$.
ii) For each fixed $t\ge 0$, the map $(\epsilon ,u)\mapsto D_u U_\epsilon (t;u)$ is continuous from $(0,\epsilon _0)\times X_+$ into $\mathcal {L}(X)$.

In the following, we prove the continuity of the map $(\epsilon ,u)\mapsto D_u U_\epsilon (t;u)$ on $\{0\} \times X_+$. To this end, we first prove the following technical lemma.

#### Lemma 7.8

Let Assumption 1.1 be satisfied and $\mu _1>0$. Let *B* be a bounded subset of $X_+$. Then for each $\tau ^*>0$ and $\tau \in (0,\tau ^*)$, the set

$$\begin{aligned} \mathcal {A}=\{D_uU_0(t;\varphi ).h: t\in [\tau ,\tau ^*],\,\varphi \in B,\,h\in B_X(0,1)\} \end{aligned}$$

is relatively compact in *X*.

#### Proof

Let *B* be a bounded subset of $X_+$, $\tau ^*>0$ and $\tau \in (0,\tau ^*)$. To prove the relative compactness of $\mathcal {A}$, we define the following map *G* on $\mathbb {R}_+\times B\times X_+$ by

7.22 $$\begin{aligned} G(t;\varphi ,u)=(b-\mu _m-2U_0(t,\varphi ))u. \end{aligned}$$

Consider the following sets

7.23 $$\begin{aligned} \{D_uU_0(t;\varphi ).h: t\in [0,\tau ^*],\,\varphi \in B,\,h\in B_X(0,1)\} \end{aligned}$$

and

7.24 $$\begin{aligned} \{G(t;\varphi ,D_uU_0(t;\varphi ).h): t\in [0,\tau ^*],\,\varphi \in B,\,h\in B_X(0,1)\}. \end{aligned}$$

By comparison principle, we have for all $t\in [0,\tau ^*]$, $\varphi \in B$ and $h\in B_X(0,1)$,

7.25 $$\begin{aligned} \Vert D_uU_0(t;\varphi ).h\Vert \le e^{b\tau ^*}\Vert h\Vert \le e^{b\tau ^*}. \end{aligned}$$

Since the function *G* defined in (7.22) is continuous on $\mathbb {R}_+\times B\times X_+$, it follows from (7.25) that the sets in (7.23) and (7.24) are bounded.

Now, let define

$$\begin{aligned} \kappa _1=\sup _{ t\in [0,\tau ^*],\,\varphi \in B,\,h\in B_X(0,1)}\Vert G(t;\varphi ,D_uU_0(t;\varphi ).h)\Vert . \end{aligned}$$

Using the properties of semigroup, for every $t\in [\tau ,\tau ^*]$, $\varphi \in B$ and $h\in B_X(0,1)$, we can derive

7.26 $$\begin{aligned} \begin{array}{lllll} D_uU_0(t;\varphi ).h& =& T_A(t)h+\int _0^tT_A(t-s)G(s;\varphi ,D_uU_0(s;\varphi ).h)\textrm{d}s\\ & =& T_A(\tau )\left( T_A(t-\tau )h+\int _0^{t-\tau }T_A(t-\tau -s)G(s;\varphi ,D_uU_0(s;\varphi ).h)\textrm{d}s\right) \\ & & +\int _{t-\tau }^tT_A(t-s)G(s;\varphi ,D_uU_0(s;\varphi ).h)\textrm{d}s.\\ \end{array} \end{aligned}$$

Let define

$$\begin{aligned} \begin{aligned} \mathcal {A}_\tau =\bigg \{T_A(\tau )&\left( T_A(t-\tau )h+\int _0^{t-\tau }T_A(t-\tau -s)G(s;\varphi ,D_uU_0(s;\varphi ).h)\textrm{d}s\right) :\\&t\in [\tau ,\tau ^*],\varphi \in B(\varphi _0,\delta ),h\in B(0,1)\bigg \}. \end{aligned} \end{aligned}$$

For every $t\in [\tau ,\tau ^*]$, $\varphi \in B$ and $h\in B_X(0,1)$, we have:

7.27 $$\begin{aligned} \begin{aligned} \Vert T_A(t-\tau ) h+&\int _0^{t-\tau }T_A(t-\tau -s)G(s;\varphi ,D_uU_0(s;\varphi ).h)\textrm{d}s\Vert \\&\le \Vert T_A(t-\tau )\Vert \Vert h\Vert +\int _0^{t-\tau }\Vert T_A(t-\tau -s)\Vert \Vert G(s;\varphi ,D_uU_0(s;\varphi ).h)\Vert \textrm{d}s\\&\le 1+(\tau ^*-\tau )\kappa _1. \end{aligned}\nonumber \\ \end{aligned}$$

Using (7.27) and the compactness of the operator $T_A(\tau )$, we conclude that the set $\mathcal {A}_\tau$ is relatively compact. Moreover, combining (7.26) and (7.27), we obtain

$$\begin{aligned} \left\| \int _{t-\tau }^tT_A(t-s)G(s;\varphi ,D_uU_0(s;\varphi ).h)\textrm{d}s\right\| \le \tau \kappa _1. \end{aligned}$$

Thus, for every $f\in \mathcal {A}$, there exists $g\in \mathcal {A}_\tau$ such that $\Vert f-g\Vert \le \tau \kappa _1$. Therefore, $\mathcal {A}$ is relatively compact. $\square$

#### Lemma 7.9

Let Assumption 1.1 be satisfied and $\mu _1>0$. Then, for each fixed $\tau \ge 0$, the map $(\epsilon ,u)\in [0,\epsilon _0]\times X_+ \mapsto D_uU_\epsilon (\tau ;u) \in \mathcal {L}(X)$ is continuous on $\{0\}\times X_+$.

#### Proof

Let $\tau \ge 0$, $\delta >0$ and $\varphi \in X_+$ be given and fixed. Define for each $\tau \ge 0$, $\psi \in B_X(\varphi ,\delta )$, and $h\in B_X(0,1)$

7.28 $$\begin{aligned} v_\epsilon (\tau ,u,h)=D_uU_\epsilon (\tau ;u).h,\ \forall \epsilon \in [0,\epsilon _0], u\in \{\varphi ,\psi \}. \end{aligned}$$

Using (7.28) it follows from Thieme (1990, Theorem 3.4), and Henry et al. (2006, Theorem 3.4.4) that

7.29 $$\begin{aligned} v_\epsilon (\tau ,\psi ,h)=T_A(\tau )h+\int _0^\tau T_A(\tau -s)D_uF_\epsilon (U_\epsilon (s;\psi )).v_\epsilon (s,\psi ,h)\textrm{d}s \end{aligned}$$

and

7.30 $$\begin{aligned} v_0(\tau ,\varphi ,h)=T_A(\tau )h+\int _0^\tau T_A(\tau -s)D_uF_0(U_0(s;\varphi )).v_0(s,\varphi ,h)\textrm{d}s. \end{aligned}$$

From (7.29) and (7.30), we obtain for all $h\in B_X(0,1)$, $\psi \in B_X(\varphi ,\delta )$ and $\epsilon \in [0,\epsilon _0]$

$$\begin{aligned} v_\epsilon (\tau ,\psi ,h)-v_0(\tau ,\varphi ,h)=W_{1,\epsilon }(\tau ,\varphi ,\psi ,h)+W_{2,\epsilon }(\tau ,\varphi ,\psi ,h) \end{aligned}$$

where we have set for each $s\in [0,\tau ]$

$$\begin{aligned} {\left\{ \begin{array}{ll} W_{1,\epsilon }(\tau ,\varphi ,\psi ,h)=\displaystyle \int _0^\tau T_A(\tau -s)\circ D_uF_\epsilon (U_\epsilon (s;\psi )).[v_\epsilon (s,\psi ,h)-v_0(s,\varphi ,h)]\textrm{d}s \\ W_{2,\epsilon }(\tau ,\psi ,\varphi ,h)=\displaystyle \int _0^\tau T_A(\tau -s)\circ [D_uF_\epsilon (U_\epsilon (s;\psi ))-D_uF_0(U_0(s;\varphi ))].\\ v_0(s,\varphi ,h)\textrm{d}s. \end{array}\right. } \end{aligned}$$

In the following, we will show that there exists a constant $M_\varphi >0$ such that for every $\psi \in B_X(\varphi ,\delta )$ and $h\in B_X(0,1)$

7.31 $$\begin{aligned} \Vert W_{1,\epsilon }(\tau ,\varphi ,\psi ,h)\Vert \le M_\varphi \displaystyle \int _0^\tau \Vert (v_\epsilon (s,\psi ,h)-v_0(s,\varphi ,h))\Vert \textrm{d}s \end{aligned}$$

and

$$\begin{aligned} \lim _{(\epsilon ,\psi )\rightarrow (0,\varphi )}\sup _{\Vert h\Vert \le 1}\Vert W_{2,\epsilon }(\tau ,\psi ,\varphi ,h)\Vert =0. \end{aligned}$$

Once established, Gronwall’s Lemma will complete the proof. The proof will be done in two steps.

**Step 1:** In this step, we aim to prove (7.31). First, note that for each $s\in [0,\tau ]$, $\psi \in B_X(\varphi ,\delta )$ and $h\in B_X(0,1)$ we have

7.32 $$\begin{aligned} &  \Vert W_{1,\epsilon }(\tau ,\psi ,\varphi ,h)\Vert \le \int _0^\tau \Vert T_A(\tau -s)\Vert _{\mathcal {L}(X)}\Vert D_uF_\epsilon (U_\epsilon (s;\psi ))\Vert _{\mathcal {L}(X)} \Vert v_\epsilon (s,\psi ,h)\nonumber \\  &  \quad -v_0(s,\varphi ,h)\Vert \textrm{d}s. \end{aligned}$$

Recall that for all $u_0\in X_+$, $t\ge 0$ and $h\in B_X(0,1)$, we have

7.33 $$\begin{aligned} \Vert T_A(t)u_0\Vert \le \Vert u_0\Vert , \end{aligned}$$

and

7.34 $$\begin{aligned} D_uF_\epsilon (u_0).h=(b-\mu _m-K_\epsilon *u_0).h-u_0(K_\epsilon *h),\ \forall \epsilon \in [0,\epsilon _0]. \end{aligned}$$

Thanks to Theorem 2.2, the set $\{U_\epsilon (s;\psi ): s\in [0,\tau ],\ \psi \in B_X(\varphi ,\delta ),\ \epsilon \in [0,\epsilon _0]\}$ is bounded. Therefore, we deduce from (7.34) that there exists a constant $M_\varphi >0$ such that for every $s\in [0,\tau ]$, $\psi \in B_X(\varphi ,\delta )$ and $h\in B_X(0,1)$

7.35 $$\begin{aligned} \Vert D_uF_\epsilon (U_\epsilon (s;\psi ))\Vert _{\mathcal {L}(X)}\le M_\varphi . \end{aligned}$$

Thus, by combining (7.32), (7.33) and (7.35), we conclude that (7.31) holds for all $\psi \in B_X(\varphi ,\delta )$ and $h\in B_X(0,1)$.

**Step 2:** In this step, we estimates the functions $W_{2,\epsilon }$. To this end, note that by using (7.33), we obtain for every $\psi \in B_X(\varphi ,\delta )$

7.36 $$\begin{aligned} \Vert W_{2,\epsilon }(\tau ,\psi ,\varphi ,h)\Vert \le \int _0^\tau \Vert [D_uF_\epsilon (U_\epsilon (s;\psi ))-D_uF_0(U_0(s;\varphi ))].v_0(s,\varphi ,h)\Vert \textrm{d}s.\nonumber \\ \end{aligned}$$

Using (7.34) and (7.36), we obtain

$$\begin{aligned} \begin{aligned} \Vert W_{2,\epsilon }(\tau ,\psi ,\varphi ,h)\Vert \le&\int _0^\tau \Vert K_\epsilon *U_\epsilon (s;\psi )-U_0(s;\varphi )\Vert \Vert v_0(s,\varphi ,h)\Vert \textrm{d}s \\&+\int _0^\tau \Vert K_\epsilon *v_0(s,\varphi ,h)-v_0(s,\varphi ,h)\Vert \Vert U_\epsilon (s,\psi )\Vert \textrm{d}s\\&+\int _0^\tau \Vert U_\epsilon (s;\psi )-U_0(s;\varphi )\Vert \Vert v_0(s,\varphi ,h)\Vert \textrm{d}s, \end{aligned} \end{aligned}$$

from where we get

$$\begin{aligned} \Vert W_{2,\epsilon }(\tau ,\psi ,\varphi ,h)\Vert \le V_{1,\epsilon }(\tau ,\psi ,\varphi ,h)+ V_{2,\epsilon }(\tau ,\psi ,\varphi ,h)+ V_{3,\epsilon }(\tau ,\psi ,\varphi ,h), \end{aligned}$$

with

$$\begin{aligned} {\left\{ \begin{array}{ll} V_{1,\epsilon }(\tau ,\psi ,\varphi ,h)=\int _0^\tau \Vert K_\epsilon *U_\epsilon (s;\psi )-U_0(s;\varphi )\Vert \Vert v_0(s,\varphi ,h)\Vert \textrm{d}s\\ V_{2,\epsilon }(\tau ,\psi ,\varphi ,h)=\int _0^\tau \Vert K_\epsilon *v_0(s,\varphi ,h)-v_0(s,\varphi ,h)\Vert \Vert U_\epsilon (s,\psi )\Vert \textrm{d}s\\ V_{3,\epsilon }(\tau ,\psi ,\varphi ,h)=\int _0^\tau \Vert U_\epsilon (s;\psi )-U_0(s;\varphi )\Vert \Vert v_0(s,\varphi ,h)\Vert \textrm{d}s. \end{array}\right. } \end{aligned}$$

Our next step is to estimate the functions $V_{1,\epsilon }$, $V_{2,\epsilon }$, and $V_{3,\epsilon }$. Before proceeding, note that by the comparison principle, we have, for all $s\in [0,\tau ]$ and $h\in B_X(0,1)$

7.37 $$\begin{aligned} \Vert v_0(s,\varphi ,h)\Vert \le e^{bs}\Vert h\Vert \le e^{bs}. \end{aligned}$$

Let $\beta \in (0,\tau )$ be given. Then for every $\psi \in B_X(\varphi ,\delta )$ and $h\in B_X(0,1)$, the functions $V_{1,\epsilon }$, and $V_{2,\epsilon }$ can be decomposed as follows:

7.38 $$\begin{aligned} V_{1,\epsilon }(\tau ,\psi ,\varphi ,h)&=\int _0^\beta \Vert K_\epsilon *U_\epsilon (s;\psi )-U_0(s;\varphi )\Vert \Vert v_0(s,\varphi ,h)\Vert \textrm{d}s \nonumber \\&\quad +\int _\beta ^\tau \Vert K_\epsilon *U_\epsilon (s;\psi )-U_0(s;\varphi )\Vert \Vert v_0(s,\varphi ,h)\Vert \textrm{d}s \end{aligned}$$

and

7.39 $$\begin{aligned} V_{2,\epsilon }(\tau ,\psi ,\varphi ,h)&= \int _0^\beta \Vert K_\epsilon *v_0(s,\varphi ,h)-v_0(s,\varphi ,h)\Vert \Vert U_\epsilon (s,\psi )\Vert \textrm{d}s \nonumber \\&\quad +\int _\beta ^\tau \Vert K_\epsilon *v_0(s,\varphi ,h)-v_0(s,\varphi ,h)\Vert \Vert U_\epsilon (s,\psi )\Vert \textrm{d}s. \end{aligned}$$

Using (7.38), we infer that for every $\psi \in B_X(\varphi ,\delta )$, $h\in B_X(0,1)$ and $\epsilon \in [0,\epsilon _0]$ we have

7.40 $$\begin{aligned}&V_{1,\epsilon }(\tau ,\psi ,\varphi ,h) = \int _0^\beta (\Vert U_\epsilon (s;\psi )\Vert +\Vert U_0(s;\varphi )\Vert )\Vert v_0(s,\varphi ,h)\Vert \textrm{d}s \nonumber \\&\quad \quad \quad \quad \quad \quad \quad \quad \quad \quad +\int _\beta ^\tau \Vert K_\epsilon *U_\epsilon (s;\psi )-U_0(s;\varphi )\Vert \Vert v_0(s,\varphi ,h)\Vert \textrm{d}s. \end{aligned}$$

Since the map $(\epsilon ,u)\mapsto K_\epsilon *u$ is continuous and the set $\{U_\epsilon (s,\psi ): s\in [\beta ,\tau ],\ \psi \in B_X(\varphi ,\delta ),\ \epsilon \in [0,\epsilon _0]\}$ is compact, for all $\zeta >0$, there exists $\bar{\epsilon }_1\in (0,\epsilon _0)$ such that for every $\psi \in B_X(\varphi ,\delta )$, $\epsilon \in (0,\bar{\epsilon }_1)$ and $h\in B_X(0,1)$, we have

7.41 $$\begin{aligned} \Vert K_\epsilon *U_\epsilon (s;\psi )-U_0(s;\varphi )\Vert \le \frac{b\zeta }{e^{b\tau }-e^{b\beta }}. \end{aligned}$$

Setting

$$\begin{aligned} M_0=\sup _{s\in [0,\tau ],\psi \in B_X(\varphi ,\delta ),\epsilon \in [0,\epsilon _0]}\Vert U_\epsilon (s;\psi )\Vert \end{aligned}$$

It follows from (7.37), (7.40) and (7.41) that for all $\psi \in B_X(\varphi ,\delta )$, $\epsilon \in (0,\bar{\epsilon }_1)$ and $h\in B_X(0,1)$,

7.42 $$\begin{aligned} V_{1,\epsilon }(\tau ,\psi ,\varphi ,h)\le 2M_0\int _0^\beta e^{b s}\textrm{d}s +\frac{b\zeta }{e^{b\tau }-e^{b\beta }}\int _\beta ^\tau e^{b s}\textrm{d}s=\frac{2M_0}{b}(e^{b\beta }-1)+\zeta .\nonumber \\ \end{aligned}$$

We now need to estimate the function $V_{2,\epsilon }$. For this purpose, using (7.39), for every $\psi \in B_X(\varphi ,\delta )$, $h\in B_X(0,1)$ and $\epsilon \in [0,\epsilon _0]$ we have

7.43 $$\begin{aligned} &  V_{2,\epsilon }(\tau ,\psi ,\varphi ,h)=2\int _0^\beta \Vert v_0(s,\varphi ,h)\Vert \Vert U_\epsilon (s,\psi )\Vert \textrm{d}s \nonumber \\ &  \quad +\int _\beta ^\tau \Vert K_\epsilon *v_0(s,\varphi ,h)-v_0(s,\varphi ,h)\Vert \Vert U_\epsilon (s,\psi )\Vert \textrm{d}s. \end{aligned}$$

Given that the set $\{v_0(s,\varphi ,h): s\in [\beta ,\tau ], h\in B_X(0,1)\}$ is compact, for all $\zeta >0$ there exist $\bar{\epsilon }_2\in [0,\epsilon _0]$ and $\bar{\delta }\in (0,\delta )$ such that for every $s\in [\beta ,\tau ]$, $\epsilon \in (0,\bar{\epsilon }_2)$, $\psi \in B_X(\varphi ,\bar{\delta })$ and $h\in B_X(0,1)$, we have

7.44 $$\begin{aligned} \Vert K_\epsilon *v_0(s,\varphi ,h)-v_0(s,\varphi ,h)\Vert \le \zeta (1+(\tau -\beta )M_0)^{-1}. \end{aligned}$$

Thus, using (7.44), it follows from (7.43) that for every $\epsilon \in (0,\bar{\epsilon }_2)$, $\psi \in B_X(\varphi ,\bar{\delta })$ and $h\in B_X(0,1)$

7.45 $$\begin{aligned} V_{2,\epsilon }(\tau ,\psi ,\varphi ,h)\le \frac{M_0}{b}(e^{b\beta }-1)+\zeta . \end{aligned}$$

Let consider the function $V_{3,\epsilon }$. Using Proposition 7.6-ii), we deduce that for all $\zeta >0$, there exist $\bar{\epsilon }_3\in (0,\epsilon _0)$, $\bar{\delta }_1\in (0,\delta )$ such that for every $s\in [\beta ,\tau ]$, $\psi \in B_X(\varphi ,\bar{\delta }_1)$, $h\in B_X(0,1)$ and $\epsilon \in (0,\bar{\epsilon }_3)$ we have

7.46 $$\begin{aligned} \Vert U_\epsilon (s;\psi )-U_0(s;\varphi )\Vert \le \frac{b\zeta }{e^{b\zeta }-1}. \end{aligned}$$

Consequently, coupling (7.37) and (7.46), we deduce that for every $s\in [\beta ,\tau ]$, $\psi \in B_X(\varphi ,\bar{\delta }_1)$, $h\in B_X(0,1)$ and $\epsilon \in (0,\bar{\epsilon }_3)$ we have

7.47 $$\begin{aligned} V_{3,\epsilon }(\tau ,\psi ,\varphi ,h)\le \zeta . \end{aligned}$$

Finally, combining (7.42), (7.45), and (7.47), we conclude that there exist $\bar{\delta }\in (0,\delta )$ and $\bar{\epsilon }\in (0,\epsilon _0)$ such that for every $\psi \in B_X(\varphi ,\delta )$, $h\in B_X(0,1)$ and $\epsilon \in (0,)$ we have

7.48 $$\begin{aligned} W_{2,\epsilon }(\tau ,\psi ,\varphi ,h) &  \le V_{1,\epsilon }(\tau ,\psi ,\varphi ,h)+V_{2,\epsilon }(\tau ,\psi ,\varphi ,h)+V_{3,\epsilon }(\tau ,\psi ,\varphi ,h)\nonumber \\  &  \le \zeta +\frac{3M_0}{b}(e^{\beta b}-1). \end{aligned}$$

Since $\zeta$ and $\beta$ are arbitrary chosen, we conclude using (7.48) that

$$\begin{aligned} \sup _{\Vert h\Vert \le 1}W_{2,\epsilon }(\tau ,\psi ,\varphi ,h)\rightarrow 0 \text { as } (\epsilon ,\psi )\rightarrow (0,\varphi ). \end{aligned}$$

This completes the proof. $\square$

#### Lemma 7.10

Let Assumption 1.1 be satisfied and $\mu _1>0$. Then the family of bounded linear operator $\{D_uU_0(t;\varphi _0)\}_{t\ge 0}$ defines a strongly continuous semigroup with negative growth bound, namely, there exists $\omega <0$ such that $r(D_uU_0(t;\varphi _0))=\exp (t\omega )$ for all $t\ge 0$ where for each $t\ge 0$, $r(D_uU_0(t;\varphi _0))$ denotes the spectral radius of $D_uU_0(t;\varphi _0)$.

#### Proof

The family $\{D_uU_0(t;\varphi _0)\}_{t\ge 0}$ is a strongly continuous semigroup generated by the linear operator $L_0: D(L_0)\subset X \rightarrow X$ given by

$$\begin{aligned} {\left\{ \begin{array}{ll} D(L_0)=\{v\in X: \,v''\in X\}\\ L_0v=Dv''+(b-\mu _m-2 \varphi _0)v. \end{array}\right. } \end{aligned}$$

Since $L_0$ is a linear elliptic operator, then we deduce by the Krein-Rutman Theorem (see Lam and Lou 2022, Theorem 1.3.6) that there exist $\omega \in \mathbb {R}$ and $\phi _0\in X_+$ and $\phi _0>0$ such that

$$\begin{aligned} {\left\{ \begin{array}{ll} L_0\phi _0=\omega \phi _0\\ \phi _0\in X,\ \phi _0>0,\ \Vert \phi _0\Vert =1. \end{array}\right. } \end{aligned}$$

Furthermore, we have for each $t\ge 0$,

7.49 $$\begin{aligned} D_uU_0(t;\varphi _0)\phi _0=e^{t\omega }\phi _0. \end{aligned}$$

Since the family $\lbrace D_uU_0(t;\varphi _0)\rbrace _{t\ge 0}$ is an analytic semigroup generated by a linear elliptic operator, then it is compact and monotone with respect to $X_+$. It follows from (7.49) and the strong form of the Krein–Rutman Theorem (see Lam and Lou 2022, Theorem B.3.2) that $r(D_uU_0(t;\varphi _0))=e^{t\omega }$ for each $t>0$. Thus, it remains to show that $\omega <0$. For this, we assume by contradiction that $\omega \ge 0$. Since $\phi _0$ and $\varphi _0$ are strictly positive and bounded, one defines

$$\begin{aligned} k^*=\inf \{k> 0: \phi _0-k\varphi _0<0\}\ge 0. \end{aligned}$$

Assume $k^*>0$ and let $z:=\phi _0-k^*\varphi _0\le 0$. Since *z* is periodic and continuous, there exists $x^*\in [0,1]$ such that $z(x^*)=0$. Thus, as *z* satisfies the following equation

7.50 $$\begin{aligned} {\left\{ \begin{array}{ll} Dz''+(b-\mu _m-\varphi _0)z=(\omega +\varphi _0)\phi _0>0\\ z\le 0\,\text { in }\, [0,1], \end{array}\right. } \end{aligned}$$

we infer by the strong maximum principle that $z\equiv 0$. Consequently, we obtain from (7.50) the contradiction $0=(\omega +\varphi _0)\phi _0>0$. This implies that $\omega <0$. $\square$

#### Proof of Theorem 2.4

Let $\delta >0$ be given and fixed. Let *B* be a bounded subset of $X_+{\setminus }\{0\}$. We define the operator $T_\epsilon$ for $(\epsilon ,u)\in [0,\epsilon _0]\times X_+$ as follows:

$$\begin{aligned} T_\epsilon (u)=U_\epsilon (1;u). \end{aligned}$$

The proof of Theorem 2.4 will be done in three steps.

**Step 1:** In this step, we show that the operator $T_\epsilon$ is a uniform contraction mapping of a bounded subset of $X_+$. Given that $D_u U_\epsilon (t;\varphi )$ exists and is continuous with respect to $(\epsilon ,u)\in [0,\epsilon _0]\times B_X(\varphi _0,\delta )$ for each $t\ge 0$ fixed, we observe from Lemma 7.3 and Lemma 7.10 that

7.51 $$\begin{aligned} T_0(\varphi _0)=\varphi _0,\ r(D_u(T_0(\varphi _0)))<1, \end{aligned}$$

$$\begin{aligned} T_0^n\psi \rightarrow \varphi _0\ \text { as } n\rightarrow \infty \text { for every } \psi \in X_+\setminus \{0\}. \end{aligned}$$

By using (7.51), it follows from Krasnosel’skiĭ (1964) that there exists a norm on $\mathcal {L}(X)$ equivalent to the operator norm defined on *X* relabeled $\Vert \cdot \Vert _{\mathcal {L}(X)}$ and which satisfies:

7.52 $$\begin{aligned} \Vert D_u T_0(\varphi _0)\Vert _{\mathcal {L}(X)}<\rho <1. \end{aligned}$$

Since the map $(\epsilon ,\varphi )\mapsto D_u T_\epsilon (\varphi )$ is continuous, we deduce from (7.52) that there is $\eta , \epsilon _1>0$ such that

7.53 $$\begin{aligned} \Vert D_u T_\epsilon (\varphi )\Vert _{\mathcal {L}(X)}<\rho \ \text {for}\ \varphi \in B_X(\varphi _0,\eta )\ \text {and}\ \epsilon \in [0,\epsilon _1]. \end{aligned}$$

Using the continuity of the map $\epsilon \mapsto T_\epsilon (\varphi _0)$, we infer there exists $\xi _0\in (0,\epsilon _1)$ such that

7.54 $$\begin{aligned} \Vert T_\epsilon (\varphi _0)-T_0(\varphi _0)\Vert \le (1-\rho )\eta ,\; \text { for }\epsilon \in [0,\xi _0]. \end{aligned}$$

Hence, using (7.53) and (7.54), we deduce that for $\varphi ,\varphi '\in B_X(\varphi _0,\eta )$ and $\epsilon \in [0,\xi _0]$, we have

7.55 $$\begin{aligned} \Vert T_\epsilon (\varphi )-T_\epsilon (\varphi ')\Vert \le \int _0^1\Vert D_u T_\epsilon (s\varphi +(1-s)\varphi ')\Vert _{\mathcal {L}(X)}\textrm{d}s\Vert \varphi -\varphi '\Vert \le \rho \Vert \varphi -\varphi '\Vert \nonumber \\ \end{aligned}$$

and

7.56 $$\begin{aligned} \begin{array}{llll} \Vert T_\epsilon (\varphi )-\varphi _0\Vert & \le & \Vert T_\epsilon (\varphi )-T_\epsilon (\varphi _0)\Vert +\Vert T_\epsilon (\varphi _0)-T_0(\varphi _0)\Vert \\ & \le & \rho \Vert \varphi -\varphi _0\Vert +(1-\rho )\eta \le \eta . \end{array} \end{aligned}$$

Combining (7.55) and (7.56), we deduce that $T_\epsilon$ is uniform contraction mapping of $\overline{B_X(\varphi _0,\eta )}$ for any $\epsilon \in [0,\xi _0]$. Consequently, there is a continuous function $\varphi :[0,\xi _0]\rightarrow \overline{B_X(\varphi _0,\eta )}$ such that

$$\begin{aligned} \varphi (0)=\varphi _0\ \text { and } T_\epsilon (\varphi (\epsilon ))=\varphi (\epsilon ). \end{aligned}$$

Furthermore, $T^m_\epsilon (\psi )\rightarrow \varphi (\epsilon )$ as $m\rightarrow \infty$ for every $\psi \in \overline{B_X(\varphi _0,\eta )}$ and $\epsilon \in [0,\xi _0]$.

**Step 2:** In this step, we prove that there exists $\xi _1:=\xi _1(B)\in (0,\xi _0)$ and $m\in \mathbb {N}$ such that for every $\epsilon \in [0,\xi _1]$ and $\psi \in C:=\overline{\bigcup _{\xi \in [0,\epsilon _0]}T_\xi (B)}$, we have

7.57 $$\begin{aligned} T^m_\epsilon (\psi )\in B_X(\varphi _0,\eta ). \end{aligned}$$

To establish (7.57), we proceed by contradiction. Suppose that there exists a sequences $\epsilon _n\in [0,\xi _0]$ and $\psi _n\in C$ such that $\epsilon _n\rightarrow 0$ and

$$\begin{aligned} \Vert T^m_{\epsilon _n}(\psi _n)-\varphi _0\Vert \ge \eta \text { for all } m\ge 0,\, n\ge 1. \end{aligned}$$

Since the set *C* is compact (cf. Proposition 7.5), there exists $\psi \in C$ such that $\psi _n\rightarrow \psi$ as $n\rightarrow +\infty$. Moreover, since $\varphi _0$ is globally attracting for $T_0$ there exists a $p\in \mathbb {N}$ such that $\Vert T_0^p(\psi )-\varphi _0\Vert <\dfrac{\eta }{2}$. Therefore, by using the continuity of map $(\epsilon ,u)\mapsto T^p_\epsilon (u)$, we conclude that $T^p_{\epsilon _n}(\psi _n)\rightarrow T_0^p(\psi )$ as $n\rightarrow \infty$. This implies that $\Vert T^p_{\epsilon _n}(\psi _n)-\varphi _0\Vert <\eta$ for all large *n* and leading to a contradiction.

**Step 3:** The aim of this step is to prove that

$$\begin{aligned} U_\epsilon (\tau ;\varphi (\epsilon ))=\varphi (\epsilon ) \text { for each } \tau \ge 0 \text { and } \epsilon \in [0,\xi _1] \end{aligned}$$

and

$$\begin{aligned} U_\epsilon (t;\psi )\rightarrow \varphi (\epsilon )\, \text { for every } \psi \in B \text { and } \epsilon \in [0,\xi _1]. \end{aligned}$$

According to Theorem 2.2, there exists $\kappa :=\kappa (B)>0$ such that for all $\psi \in B$ and $\epsilon \in [0,\epsilon _0]$, $T_\epsilon (\psi )\in B_X(0,\kappa )$. Consequently, we can deduce that

7.58 $$\begin{aligned} T_\epsilon ^2(\psi )\in T_\epsilon (B_X(0,\kappa ))\ \text { for every } \psi \in B \text { and } \epsilon \in [0,\epsilon _0]. \end{aligned}$$

Using the result of step 2, we deduce that there exist $\xi _1:=\xi _1(\kappa )\in (0,\xi _0)$ and $m\in \mathbb {N}$ such that for any $\epsilon \in [0,\xi _1]$ and $\psi _1\in \overline{\bigcup _{\xi \in [0,\epsilon _0]}T_\xi (B_X(0,\kappa ))}$, the following hold:

7.59 $$\begin{aligned} T^m_\epsilon (\psi _1)\in B_X(\varphi _0,\eta ). \end{aligned}$$

Combining (7.58) and (7.59), we deduce that for all $\psi \in B$ and $\epsilon \in [0,\xi _1]$, we have

7.60 $$\begin{aligned} T^2_\epsilon (\psi )\in T_\epsilon (B_X(0,\kappa ))\subset \overline{\bigcup _{\xi \in [0,\epsilon _0]}T_\xi (B_X(0,\kappa ))}\ \text { and }\ T_\epsilon ^{m+2}(\psi )\in B(\varphi _0,\eta ). \end{aligned}$$

Hence, using (7.60) and the result of Step 1, we deduce that for each $\psi \in B$ and $\epsilon \in [0,\xi _1]$ we have

7.61 $$\begin{aligned} T^n_\epsilon (\psi )= U_\epsilon (n;\psi )\rightarrow \varphi (\epsilon ) \text { as } n\rightarrow \infty . \end{aligned}$$

Let $\psi ^*\in B$ and $\tau \ge 0$ be given. Setting $\psi :=U_\epsilon (\tau ;\psi ^*)$, we obtain from (7.61)

7.62 $$\begin{aligned} U_\epsilon (\tau ;U_\epsilon (n;\psi ^*))= U_\epsilon (n+\tau ;\psi ^*)\rightarrow \varphi (\epsilon )\ \text { as } n\rightarrow \infty \end{aligned}$$

Next, we infer from (7.61) that $U_\epsilon (n;\psi ^*) \rightarrow \varphi (\epsilon )$ when $n\rightarrow +\infty$ so that $U_\epsilon (\tau ;U_\epsilon (n;\psi ^*))\rightarrow U_\epsilon (\tau ;\varphi (\epsilon ))$, when $n \rightarrow +\infty$. Hence, letting *n* goes to $+\infty$ in the left hand side of (7.62) we obtain

$$\begin{aligned} U_\epsilon (\tau ;\varphi (\epsilon ))=\varphi (\epsilon )\ \text { for each } \tau \ge 0 \text { and } \epsilon \in [0,\xi _1]. \end{aligned}$$

Now, we claim that $U_\epsilon (t;\psi )\rightarrow \varphi (\epsilon )$ as $t\rightarrow \infty$ for each $\psi \in B$ and $\epsilon \in [0,\xi _1]$. If not, there exist $\psi \in B$, $\epsilon \in [0,\xi _1]$, $\eta >0$ and $t_n\rightarrow \infty$ such that

7.63 $$\begin{aligned} \Vert U_\epsilon (t_n;\psi )-\varphi (\epsilon )\Vert \ge \eta \text { for } n\ge 1. \end{aligned}$$

For each $n\in \mathbb {N}$, denotes by $m_n\in \mathbb {N}$ and $\tau _n \in [0,1)$ the real numbers such $t_n=m_n+\tau _n$. Since the sequence $(\tau _n)\subset [0,1)$ is bounded, it admits a sub-sequence, denoted by $(\tau _n)$ which converges to $\tau \in [0,1]$. Thus, by using (7.61) and the continuity of the semiflow with respect to the state variable, we obtain, along a subsequence

$$\begin{aligned} U_\epsilon (t_n;\psi )=U_\epsilon (m_n+\tau _n;\psi )=U_\epsilon (\tau _n;U_\epsilon (m_n;\psi ))\underset{n \rightarrow +\infty }{\longrightarrow } U_\epsilon (\tau ;\varphi (\epsilon ))=\varphi (\epsilon ) \end{aligned}$$

which leads to a contradiction by letting *n* goes to $+\infty$ in (7.63). Therefore, we conclude that

$$\begin{aligned} U_\epsilon (t;\psi )\rightarrow \varphi (\epsilon )\;\text {as}\;t\rightarrow +\infty ,\, \text { for }\psi \in B,\, \epsilon \in [0,\xi _1]. \end{aligned}$$

$$\square $$

## Proof of Theorem 3.2: disease dynamics

In this section, we study the uniform persistence of the disease for (1.1) and (1.2) with $\Lambda >0$, $\mu _h>0$. Let us first recall that the total populations of mosquitoes and humans are governed by the following system

8.1 $$\begin{aligned} {\left\{ \begin{array}{ll} \dfrac{\textrm{d}N_h(t)}{\textrm{d}t}=\Lambda -\mu _hN_h(t)-\gamma _2 I_h(t),\\ \dfrac{\partial N_m(t,x)}{\partial t}=D\partial _{xx} N_m(t,x)+bN_m(t,x)\\ \qquad \qquad \qquad \quad -N_m(t,x)\left[ \mu _m(x)+\int _0^{1} K_\epsilon (x-y)N_m(t,y)\textrm{d}y\right] . \end{array}\right. } \end{aligned}$$

Since $\mathcal {T}_0>1$ we infer from Theorem 2.4 that there exist $\xi _0\in [0,\epsilon _0]$ and a continuous map $\bar{S}_m^\epsilon :[0,\xi _0]\rightarrow X_+$ such that for each $\epsilon \in [0,\xi _0]$, $\bar{S}_m^\epsilon$ is an equilibrium state of the $N_m$-equation of (8.1) with $\bar{S}_m^\epsilon \in \text {int}(X_+)$. To prove the uniform persistence of the disease, let $\Omega \subset Z\times B$ be a bounded and closed subset with $B \subset X_+\times X_+$. Consider the set

8.2 $$\begin{aligned} \mathcal {M}_\Omega =\left\{ (S_h,I_h,S_m,I_m)\in \Omega : I_h+\Vert I_m\Vert >0\right\} \; \text {and}\; \partial \mathcal {M}_\Omega =\Omega \setminus \mathcal {M}_\Omega . \end{aligned}$$

Since $B \subset X_+\times X_+$, Theorem 2.4 ensures that there exists $\xi _1:=\xi _1(B)$ with $\xi _1\in [0,\xi _0]$ such that for each $\epsilon \in [0,\xi _1]$ and $N_m(0,\cdot )=S_{0\,m}+I_{0\,m}$ with $(S_{0\,m},I_{0\,m})\in B$ we have

$$\begin{aligned} N_m(t,\cdot )=S_m(t,\cdot )+I_m(t,\cdot )\rightarrow \bar{S}_m^\epsilon ,\ \text {as}\ t\rightarrow \infty . \end{aligned}$$

In the rest of this section, $\epsilon$ is fixed in $[0,\xi _1]$. Denote by $\bar{E}(\epsilon )$ the nontrivial disease free-equilibrium state of (1.1) and (1.2) that is

$$\begin{aligned} \bar{E}(\epsilon )=({\bar{S}}_h,0,{\bar{S}}_m^\epsilon ,0). \end{aligned}$$

### Technical materials

In this section, we explore the properties of the following system parameterized by $\tau \in [0,1)$

8.3 $$\begin{aligned} {\left\{ \begin{array}{ll} \dfrac{\textrm{d}u(t)}{\textrm{d}t}=(1-\tau )\displaystyle \int _0^1p(y)v(t,y)\textrm{d}y-(\gamma _1+\gamma _2+\mu _h)u(t) \\ \dfrac{\partial v(t,x)}{\partial t}=(1-\tau )\sigma (x)\dfrac{\bar{S}_m^\epsilon (x)}{\bar{S}_h}u(t)+D\partial _{xx}v(t,x)\\ \qquad \qquad \qquad -v(t,x)\left[ \mu _m(x)+(1+\tau )\int _0^1K_\epsilon (x-y)\bar{S}_m^\epsilon (y)\textrm{d}y\right] ,\ x\in \mathbb {R}, \end{array}\right. } \end{aligned}$$

with initial conditions $u(0)=u_0 \in \mathbb {R}$ and $v(0,\cdot )=v_0 \in X$. Note that when $\tau =0$, the later systems correspond to the linearization of the infective components of (1.1) and (1.2) around the equilibrium state $\bar{E}(\epsilon )$. More precisely, we deal with the stability and instability of the zeros solution of (1.1) and (1.2) in terms of a threshold $\mathcal {R}_{\tau ,\epsilon }$. To define the threshold $\mathcal {R}_{\tau ,\epsilon }$ we proceed as in Sect. 3.2. Let *A* be the linear operator defined in (2.2). Let $\mathcal {F}_\tau : \mathbb {R}\times X\rightarrow X$ and $\mathcal {V}_\tau : \mathbb {R}\times D(A)\rightarrow X$ defined for each $\tau \in [0,1]$ by

$$\begin{aligned} \mathcal {F}_\tau \begin{pmatrix} u\\ v \end{pmatrix}=\begin{pmatrix} (1-\tau )\int _0^1p(y)v(y)\textrm{d}y\\ (1-\tau )\sigma (\cdot )\dfrac{\bar{S}_m^\epsilon (\cdot )}{{\bar{S}}_h}u \end{pmatrix} \end{aligned}$$

and

$$\begin{aligned} \mathcal {V}_\tau \begin{pmatrix} u\\ v \end{pmatrix}=\begin{pmatrix} -(\gamma _1+\gamma _2+\mu _h)u\\ L_{\tau ,\epsilon }v \end{pmatrix} \end{aligned}$$

where

8.4 $$\begin{aligned} L_{\tau ,\epsilon }v:=D\partial _{xx} v-(\mu _m+(1+\tau ) q_{\epsilon }) v=L_{\epsilon }v-\tau q_\epsilon v,\ \forall v \in D(A). \end{aligned}$$

and $L_{\epsilon }$ is the linear operator defined in (3.8). Using the same arguments as in Sect. 3.2 one has the following relationship

8.5 $$\begin{aligned} \text {sgn}(\mathcal {R}_{\tau ,\epsilon }-1)=\text {sgn}(\textsf{s}(\mathcal {F}_\tau +\mathcal {V}_\tau )) \end{aligned}$$

with

$$\begin{aligned} \mathcal {R}_{\tau ,\epsilon }:=\textsf{r}(\mathcal {F}_\tau (-\mathcal {V}_\tau )^{-1})=\dfrac{(1-\tau )^2}{(\gamma _1+\gamma _2+\mu _h)}\int _0^1p(y)\left( \int _0^\infty T_{L_{\tau ,\epsilon }}(t)\frac{\bar{S}_m^\epsilon (\cdot )}{\bar{S}_h}\sigma \textrm{d}t\right) (y)\textrm{d}y \end{aligned}$$

and $\{T_{L_{\tau ,\epsilon }}(t)\}_{t\ge 0}$ is the semigroup generated by the linear operator $L_{\tau ,\epsilon }$. It is worth noting that $L_{\tau ,\epsilon }$ constitutes a bounded perturbation of a generator of an analytic semigroup, which implies, according to Pazy (2012), that $\{T_{L_{\tau ,\epsilon }}(t)\}_{t\ge 0}$ forms an analytic semigroup. Hence, we can readily establish that $\mathcal {F}_\tau +\mathcal {V}_\tau$ generates an analytic semigroup as it represents a bounded perturbation of a generator of an analytic semigroup. Consequently $\textsf{s}(\mathcal {F}_\tau +\mathcal {V}_\tau )=\omega (\mathcal {F}_\tau +\mathcal {V}_\tau )$ with $\omega (\mathcal {F}_\tau +\mathcal {V}_\tau )$ the growth bound of the semigroup generated by $\mathcal {F}_\tau +\mathcal {V}_\tau$. From the above discussions and (8.5), we obtain the following sign equality

$$\begin{aligned} \text {sgn}(\mathcal {R}_{\tau ,\epsilon }-1)=\text {sgn}(\textsf{s}(\mathcal {F}_\tau +\mathcal {V}_\tau ))=\text {sgn}(\omega (\mathcal {F}_\tau +\mathcal {V}_\tau )). \end{aligned}$$

Next, using (8.4) one has $T_{L_{\tau ,\epsilon }}(t)u_0\le T_{L_{\epsilon }}(t)u_0$ for all $t\ge 0$, $u_0\in X_+$ and

$$\begin{aligned} \lim _{\tau \rightarrow 0^+} T_{L_{\tau ,\epsilon }}(t)u_0=T_{L_{\epsilon }}(t)u_0 \end{aligned}$$

we obtain by a Lebesgue-dominated convergence theorem that

$$\begin{aligned} \lim _{\tau \rightarrow 0^+} \mathcal {R}_{\tau ,\epsilon }=\mathcal {R}_{0,\epsilon }=\dfrac{1}{(\gamma _1+\gamma _2+\mu _h)}\int _0^1p(y)\left( \int _0^\infty T_{L_{\epsilon }}(t)\frac{\bar{S}_m^\epsilon (\cdot )}{\bar{S}_h}\sigma \textrm{d}t\right) (y)\textrm{d}y. \end{aligned}$$

### Uniform persistence of the disease

In this subsection, we will discuss the uniformly strongly persistence of (1.1) and (1.2) in a sense that will be defined later. But first, we are interested in the uniform weakly persistent of model (1.1) and (1.2).

#### Lemma 8.1

Let Assumption 1.1 be satisfied, and assume that $\mu _1>0$. If $\mathcal {R}_{0,\epsilon }>1$, then there exists $\zeta ^*:=\zeta ^*(\Omega )>0$ such that for every $( S_{0\,h},I_{0\,h},S_{0\,m},I_{0\,m})\in \mathcal {M}_\Omega$ with $N_{0m}\not \equiv 0$, we have

8.6 $$\begin{aligned} \limsup _{t\rightarrow +\infty }(I_h(t)+\Vert I_m(t,\cdot )\Vert )\ge \zeta ^*, \end{aligned}$$

where we denote by $(S_h,I_h,S_m,I_m)$ the corresponding solution of (1.1) and (1.2) associated with the initial data $( S_{0\,h},I_{0\,h},S_{0\,m},I_{0\,m})$, and $\mathcal {M}_\Omega$ is the set defined in (8.2).

#### Proof

Assume that $\mathcal {R}_{0,\epsilon }>1$. Therefore, we can fix $\tau \in (0,1)$ small enough such that $\mathcal {R}_{\tau ,\epsilon }>1$. To establish (8.6), we argue by contradiction, and assuming that there exist a sequence $\{ (S^n_{0\,h},I^n_{0\,h},S^n_{0\,m},I^n_{0\,m})\}_{n\in \mathbb {N}}\subset \mathcal {M}_\Omega$ where $S^n_{0\,m}+I^n_{0\,m}\not \equiv 0$ for any $n\in \mathbb {N}$ and $(t_n)\subset \mathbb {R}_+$ with $t_n\rightarrow +\infty$ as $n\rightarrow +\infty$ such that

8.7 $$\begin{aligned} {\left\{ \begin{array}{ll} I_h^n(t)\le \frac{1}{n+1},\ \forall t\ge t_n,\ n\in \mathbb {N}\\ \Vert I^n_m(t,\cdot )\Vert \le \frac{1}{n+1},\,\forall t\ge t_n,\ n\in \mathbb {N}. \end{array}\right. } \end{aligned}$$

Here, for each $n\in \mathbb {N}$, $(S^n_h,I_h^n, S^n_m,I_m^n)$ denotes the corresponding the solution of (1.1) associated with the initial data $(S^n_{0h},I^n_{0h},S^n_{0m},I^n_{0m})$. Let us now examine the translated solution $\widetilde{S}^n_h(t)=S^n_h(t+t_n)$, $\widetilde{I}_h^n(t)=I_h^n(t+t_n)$, $\widetilde{S}_m^n(t,\cdot )=S_m^n(t+t_n,\cdot )$ and $\widetilde{I}_m^n(t,\cdot )=I_m^n(t+t_n,\cdot )$ for all $t\ge -t_n$. Thus, for each $n\in \mathbb {N}$, $t\ge -t_n$ and $x\in [0,1]$, we have

8.8 $$\begin{aligned} {\left\{ \begin{array}{ll} \frac{\textrm{d}\widetilde{S}_h^n(t)}{\textrm{d}t}=\Lambda -\dfrac{\widetilde{S}_h^n(t)}{\widetilde{N}_h^n(t)}\displaystyle \int _0^{1} p(y)\widetilde{I}_m^n(t,y)\textrm{d}y+\gamma _1\widetilde{I}_h^n(t)-\mu _h\widetilde{S}_h^n(t), \\ \frac{\textrm{d}\widetilde{I}_h^n(t)}{\textrm{d}t}= \dfrac{\widetilde{S}_h^n(t)}{\widetilde{N}_h^n(t)}\displaystyle \int _0^{1} p(y)\widetilde{I}_m^n(t,y)\textrm{d}y -(\gamma _1+\gamma _2+\mu _h)\widetilde{I}_h^n(t), \end{array}\right. } \end{aligned}$$

and

8.9 $$\begin{aligned} {\left\{ \begin{array}{ll} \dfrac{\partial \widetilde{S}_m^n(t,x)}{\partial t}=-\sigma (x)\dfrac{\widetilde{I}_h^n(t)}{\widetilde{N}_h^n(t)}\widetilde{S}_m^n(t,x)+D\partial _{xx} \widetilde{S}_m^n(t,x)+b\widetilde{N}_m^n(t,x)\\ \qquad \qquad \qquad \quad -\widetilde{S}_m^n(t,x)\left[ \mu _m(x)+\int _0^{1} K_\epsilon (x-y)\widetilde{N}_m^n(t,y)\textrm{d}y\right] ,\\ \dfrac{\partial \widetilde{I}_m^n(t,x)}{\partial t} =\sigma (x)\dfrac{\widetilde{I}_h^n(t)}{\widetilde{N}_h^n(t)}\widetilde{S}_m^n(t,x)+D\partial _{xx} \widetilde{I}_m^n(t,x)\\ \qquad \qquad \qquad \quad -\widetilde{I}_m^n(t,x)\left[ \mu _m(x)+\int _0^{1}K_\epsilon (x-y)\widetilde{N}_m^n(t,y)\textrm{d}y\right] , \end{array}\right. } \end{aligned}$$

with $$\widetilde{N}^n_h(t)=\widetilde{S}_h^n(t)+\widetilde{I}_h^n(t)$$ and $$\widetilde{N}_m^n(t,\cdot )=\widetilde{S}_m^n(t,\cdot )+\widetilde{I}_m^n(t,\cdot )$$.

The rest of the proof is divided into two steps.

**Step 1:** In this step, we prove that

$$\begin{aligned} \widetilde{S}^n_h(t)\rightarrow {\bar{S}}_h \, \text { and }\, \widetilde{S}^n_m(t,\cdot )\rightarrow \bar{S}_m^\epsilon \end{aligned}$$

as $n\rightarrow +\infty$ globally uniformly for $t\in \mathbb {R}_+$. Thanks to (8.7), both sequences $\widetilde{I}_h^n(t)$ and $\widetilde{I}_m^n(t,\cdot )$ converge to 0 as $n\rightarrow +\infty$ uniformly for $t\in \mathbb {R}_+$. As a consequence, to obtain the global uniform convergences of the sequence $(\widetilde{S}_h^n(t))_n$ to ${\bar{S}}_h$ (resp. $(\widetilde{S}_m^n(t,\cdot ))_n$ to ${\bar{S}}_m^\epsilon$), it suffices to show that the sequence $(\widetilde{N}^n_h(t))_n$ (resp. $(\widetilde{N}_m^n(t,\cdot ))_n$) converges to ${\bar{S}}_h$ (resp. ${\bar{S}}_m^\epsilon$) as $n\rightarrow +\infty$ globally uniformly for $t\in \mathbb {R}_+$.

We first prove the convergence of the sequence $(\widetilde{S}_h^n(t))_n$. To this end, let $\nu >0$ be any positive constant. Considering the $\widetilde{N}^n_h-$equation, we obtain the following inequalities

$$\begin{aligned} \dfrac{\textrm{d}\widetilde{N}_h^n(t)}{\textrm{d}t}\le \Lambda -\mu _h\widetilde{N}_h^n(t), \end{aligned}$$

and by integrating from $-t_n$ to *t*, one obtains

$$\begin{aligned} \widetilde{N}_h^n(t)\le N_h^n(0)e^{-\mu _h(t_n+t)}+\frac{\Lambda }{\mu _h}\left( 1-e^{-\mu _h(t_n+t)}\right) \le N_h^n(0)e^{-\mu _h t_n}+\frac{\Lambda }{\mu _h}\left( 1+e^{-\mu _ht_n}\right) \end{aligned}$$

from where the boundedness of the sequence $(N_h^n(0))$ implies that there exists $N_0>0$ such that

8.10 $$\begin{aligned} \widetilde{N}_h^n(t)\le \dfrac{\Lambda }{\mu _h}+\nu ,\ \forall n\ge N_0,\ \forall t\ge 0. \end{aligned}$$

Since $(\widetilde{I}^n_h)_n$ converges to 0 as $n\rightarrow +\infty$ uniformly on $\mathbb {R}_+$, there exists $N_1\in \mathbb {N}$ such that for all $n\ge N_1$, we have

$$\begin{aligned} \dfrac{\textrm{d}\widetilde{N}_h^n(t)}{\textrm{d}t}\ge \Lambda -\mu _h\widetilde{N}_h^n(t)-\dfrac{\nu \mu _h}{2},\ \forall n\ge N_1, t\ge 0. \end{aligned}$$

Therefore, we obtain for all $n\ge N_0+N_1$ and $t\ge 0$

$$\begin{aligned} \widetilde{N}_h^n(t)\ge \dfrac{\Lambda }{\mu _h}+\left( N_h^n(0)-\dfrac{\Lambda }{\mu _h}\right) e^{-\mu _h(t+t_n)}-\frac{\nu }{2}(1- e^{-\mu _h(t+t_n)}). \end{aligned}$$

and since the sequence $(N^n_h(0))$ is bounded it follows that there exists $N_2\ge N_0+N_1$ such that

8.11 $$\begin{aligned} \widetilde{N}_h^n(t)\ge \dfrac{\Lambda }{\mu _h}-\nu ,\ \forall t\ge 0,\ n\ge N_2. \end{aligned}$$

From (8.10) and (8.11) we deduce that $\widetilde{N}_h^n(t)$ converge to ${\bar{S}}_h=\Lambda /\mu _h$ as $n\rightarrow +\infty$ uniformly for $t\in \mathbb {R}_+$. Consequently, $\widetilde{S}_h^n(t)$ converges to ${\bar{S}}_h$ as $n\rightarrow +\infty$ uniformly in $t\in \mathbb {R}_+$.

Next, we show that $\widetilde{S}_m^n(t,\cdot )$ converges to $\bar{S}_m^\epsilon$ as $n\rightarrow +\infty$ uniformly for $t\in \mathbb {R}_+$. Note that since the sequence $( \widetilde{S}_m^n(t,\cdot )+\widetilde{I}_m^n(t,\cdot ))_n$ is bounded uniformly in $n\in \mathbb {N}$ and $t\ge 0$, we can deduce by the standard parabolic estimates that it has a sub-sequence which converges locally uniformly in times to a nonnegative function in $\mathcal {C}^{1,2}(\mathbb {R}\times [0,1])$. Thus, according to Theorem 2.4 and the continuity of the maps $t\mapsto \widetilde{S}_m^n(t,\cdot )+\widetilde{I}_m^n(t,\cdot )$ for each $n\ge 0$, there exists $\xi _1(\Omega )\in (0,\epsilon _0)$ such that for each $\epsilon \in [0,\xi _1]$, we have

8.12 $$\begin{aligned} \widetilde{S}_m^n(t,\cdot )+\widetilde{I}_m^n(t,\cdot )\rightarrow \bar{S}_m^\epsilon \, \text {as}\, n\rightarrow +\infty , \end{aligned}$$

where for each $\epsilon \in [0,\xi _1]$, $\bar{S}_m^\epsilon (\cdot )$ is a strictly positive function belonging to $X_+$.

Let $\epsilon ^*>0$ be fixed. Then for each $n\in \mathbb {N}$, we have

8.13 $$\begin{aligned} \Vert \widetilde{S}_m^n(t,\cdot )-\bar{S}_m^\epsilon \Vert \le \Vert \widetilde{S}_m^n(t,\cdot )+\widetilde{I}_m^n(t,\cdot )-\bar{S}_m^\epsilon \Vert +\Vert \widetilde{I}_m^n(t,\cdot ) \Vert . \end{aligned}$$

Thus, by using (8.7) and (8.12) we infer from (8.13) that there exists $n_0\in \mathbb {N}$ such that for all $n\ge n_0$, one has

$$\begin{aligned} \Vert \widetilde{S}_m^n(t,\cdot )-\bar{S}_m^\epsilon \Vert \le \epsilon ^*, \end{aligned}$$

for any *t* in a compact subset of $\mathbb {R}_+$. This completes the proof.

**Step 2:** In this step, we demonstrate that both sequences $(\widetilde{I}^n_h)_n$ and $(\widetilde{I}_m^n)_n$ are unbounded. Thanks to step 1, there exists $n_0\in \mathbb {N}$ such that for all $n\ge n_0$, $t\ge 0$ and $x\in [0,1]$, we have

$$\begin{aligned} \left\{ \begin{array}{llll} \dfrac{\textrm{d}\widetilde{I}_h^n(t)}{\textrm{d}t}& \ge & (1-\tau )\int _0^1p(y)\widetilde{I}_m^n(t,y)\textrm{d}y-(\gamma _1+\gamma _2+\mu _h)\widetilde{I}_h^n(t) \\ \\ \partial _t\widetilde{I}_m^n(t,x)& \ge & (1-\tau )\frac{\mu _h}{\Lambda }\sigma (x)\bar{S}_m^\epsilon (x)\widetilde{I}_h^n(t)+D\partial _{xx}\widetilde{I}_m^n(t,x)\\ & & -\widetilde{I}_m^n(t,x)\left( \mu _m(x)+(1+\tau )\int _0 ^1K_\epsilon (x-y)\bar{S}_m^\epsilon (y)\textrm{d}y\right) . \end{array}\right. \end{aligned}$$

Consider the functions $\widetilde{u}$ and $\widetilde{v}$ define by

$$\begin{aligned} \widetilde{u}(t)=e^{t\textsf{s}(\mathcal {F}_\tau +\mathcal {V}_\tau )}u_\tau \text { and } \widetilde{v}(t,x)=e^{t\textsf{s}(\mathcal {F}_\tau +\mathcal {V}_\tau )}v_\tau (x) \end{aligned}$$

where $$\textsf{s}(\mathcal {F}_\tau +\mathcal {V}_\tau )$$ is the spectral bounded of $$\mathcal {F}_\tau +\mathcal {V}_\tau $$ and $$(u_\tau ,v_\tau )$$ the associated eigenfunction. Note that for any $$\alpha ^*>0$$, $$\alpha ^*(\widetilde{u},\widetilde{v})$$ satisfies equation (8.3). Since $$I_h^n(0)+I_m^n(0,\cdot )> 0$$ for every $$n\in \mathbb {N}$$, then we have $$\widetilde{I}_h^n(t)>0$$ and $$\widetilde{I}_m^n(t,\cdot )>0$$ for all $$t>0$$. Thus there exists $$\alpha ^{**}>0$$ such that $$\alpha ^{**}\widetilde{u}<\widetilde{I}^n_h(0)$$ and $$\alpha ^{**}\widetilde{v}<\widetilde{I}^n_m(0,\cdot )$$. So, by comparison principle, we have for all $$n\ge n_0$$, $$t\ge 0$$ and $$x\in [0,1]$$

$$\begin{aligned} \alpha ^{**}\widetilde{u}(t)<\widetilde{I}^n_h(t)\text { and } \alpha ^{**}\widetilde{u}(t,x)<\widetilde{I}^n_h(t,x). \end{aligned}$$

Hence, since $\textsf{s}(\mathcal {F}_\tau +\mathcal {V}_\tau )>0$, we obtain for all $n\ge n_0$

$$\begin{aligned} \lim _{t\rightarrow +\infty }\widetilde{I}_h^n(t)=+\infty \; \text { and } \; \lim _{t\rightarrow +\infty }\Vert \widetilde{I}_m^n(t,\cdot )\Vert =+\infty . \end{aligned}$$

This contradicts the boundedness of the solution. The proof is completed. $\square$

#### Theorem 8.2

Let Assumption 1.1 be satisfied, and assume that $$\mu _1>0$$. If $$\mathcal {R}_{0,\epsilon }>1$$ then for every bounded subset $$\Omega $$ of $$Y_+$$, there exists $$\zeta ^{**}:=\zeta ^{**}(\Omega )>0$$ such that for every $$(S_{0\,h},I_{0\,h},S_{0\,m},I_{0\,m})\in \mathcal {M}_\Omega $$ with $$N_{0\,m}\not \equiv 0$$, we have

8.14 $$\begin{aligned} \liminf _{t\rightarrow +\infty } (I_h(t)+\Vert I_m(t,\cdot )\Vert )\ge \zeta ^{**}, \end{aligned}$$

where $(S_h,I_h,S_m,I_m)$ denotes the solution of (1.1) and (1.2) with the initial data $(S_{0\,h},I_{0\,h},S_{0\,m},I_{0\,m})$ and $\mathcal {M}_\Omega$ is the set defined in (8.2).

#### Proof

To establish (8.14), we proceed by contradiction. Assume that for all $n\in \mathbb {N}$ there exists a sequences $\{(S_{0\,h}^n,I_{0\,h}^n,S_{0\,m}^n,I_{0\,m}^n)\}_{n\in \mathbb {N}}\subset \mathcal {M}_\Omega$ with $N_{0m}^n:=S_{0m}^n+I_{0m}^n\not \equiv 0$ such that

8.15 $$\begin{aligned} \liminf _{t\rightarrow +\infty }(I_h^n(t)+ \Vert I_m^n(t,\cdot )\Vert )\le \dfrac{1}{n+1}. \end{aligned}$$

Thanks to Lemma 8.1, there exists $\zeta ^*:=\zeta ^{*}(\Omega )>0$ such that for each $n\in \mathbb {N}$ we have

8.16 $$\begin{aligned} \limsup _{t\rightarrow +\infty }(I_h^n(t)+ \Vert I_m^n(t,\cdot )\Vert )\ge \zeta ^{*}. \end{aligned}$$

Hence, combining (8.15) and (8.16), we infer that there exists a sequences $(t_n)$ with $t_n\rightarrow +\infty$ as $n\rightarrow +\infty$ and $(s_n)\subset (0,+\infty )$ such for all $n\in \mathbb {N}$, one has

8.17 $$\begin{aligned} \left\{ \begin{array}{lllll} I^n_h(t_n)+\Vert I^n_m(t_n,\cdot )\Vert =\dfrac{\zeta ^{*}}{2} \\ I^n_h(t)+\Vert I^n_m(t,\cdot )\Vert \le \dfrac{\zeta ^{*}}{2},\; \forall t\in [t_n,t_n+s_n)\\ I^n_h(t_n+s_n)+\Vert I^n_m(t_n+s_n,\cdot )\Vert \le \dfrac{2}{n+1}. \end{array}\right. \end{aligned}$$

Now, let us set $\epsilon ^*=\frac{1}{2}\inf _{x\in [0,1]}\bar{S}^\epsilon _m(x)$. According to Theorem 2.4, these is $\xi :=\xi (\Omega )>0$ such that for all $n\in \mathbb {N}$ there exists $T_n\gg 1$ such that for all $t\ge 0$ and $\epsilon \in (0,\xi )$, we have

8.18 $$\begin{aligned} \Vert N_m^n(t+T_n,\cdot )-\bar{S}_m^\epsilon (\cdot )\Vert <\epsilon ^*. \end{aligned}$$

Consider the functions defined for all $t\ge 0$ and $n\in \mathbb {N}$ as follows

$$\begin{aligned} {\left\{ \begin{array}{ll} \hat{S}_h^n(t)=S_h^n(t+t_n+T_n),\ \hat{I}_h^n(t)=I_h^n(t+t_n+T_n),\\ \hat{S}_m^n(t,\cdot )=S_m^n(t+t_n+T_n,\cdot ),\ \hat{I}_m^n(t,\cdot )=I_m^n(t+t_n+T_n,\cdot ). \end{array}\right. } \end{aligned}$$

Note that due to the compactness of Semiflow generates by (1.1) and (1.2), the sequences $\hat{S}_h^n$, $\hat{I}_h^n$, $\hat{S}_m^n$ and $\hat{I}_m^n$ converges locally uniformly to $S_h^\infty$, $I_h^\infty$, $S_m^\infty$ and $I_m^\infty$, respectively.

We claim that $(s_n)$ has a sub-sequence that converges to $+\infty$ when *n* goes to $+\infty$. Suppose this is not the case. Then, there exists a sub-sequence $(s_{n_k})$ of $(s_n)$ that converges to $s^*\in \mathbb {R}_+$ when *k* goes to $+\infty$. From (8.17), we obtain

8.19 $$\begin{aligned} {\left\{ \begin{array}{ll} I^{n_k}_h(t_{n_k})+\Vert I^{n_k}_m(t_{n_k},\cdot )\Vert =\dfrac{\zeta ^{*}}{2} \\ I^{n_k}_h(t_{n_k}+s_{n_k})+\Vert I^{n_k}_m(t_{n_k}+s_{n_k},\cdot )\Vert \le \dfrac{2}{n+1}. \end{array}\right. } \end{aligned}$$

Letting $k\rightarrow +\infty$ in (8.19) we obtain

8.20 $$\begin{aligned} {\left\{ \begin{array}{ll} I_h^\infty (0)+\Vert I_m^\infty (0,\cdot )\Vert =\dfrac{\zeta ^{*}}{2} \\ I_h^\infty (s^*)=0 \, \text {and} \, I_m^\infty (s^*,\cdot )\equiv 0. \end{array}\right. } \end{aligned}$$

Thus, we infer from (8.20) that $I_h^\infty (t)=0$ and $I_m^\infty (t,x)\equiv 0$ for all $t>0$ and $x\in [0,1]$, which yields a contradiction.

Next, since there exists the sequence $(s_{n_k})\subset (0,\infty )$ with $s_{n_k}\rightarrow +\infty$ as $k\rightarrow +\infty$, we infer from (8.17) that

8.21 $$\begin{aligned} {\left\{ \begin{array}{ll} I^\infty _h(0)+\Vert I^\infty _m(0,\cdot )\Vert = \dfrac{\zeta ^{*}}{2}>0\\ I^\infty _h(t)+\Vert I^\infty _m(t,\cdot )\Vert \le \dfrac{\zeta ^{*}}{2},\, \forall t\ge 0, \end{array}\right. } \end{aligned}$$

and from (8.18), we obtain

8.22 $$\begin{aligned} \widetilde{N}_m^\infty (0,\cdot )\ge \epsilon ^*>0. \end{aligned}$$

This completes the proof, since (8.21) and (8.22) contradict Lemma 8.1. $\square$
